# Supplementary material for: Associations between maternal characteristics and pharmaceutical treatment of gestational diabetes: an analysis of the UK Born in Bradford (BiB) cohort study
Source: BMJ Open. 2021 Nov 3;11(11):e053753. doi: 10.1136/bmjopen-2021-053753 (PMC8572403; doi:10.1136/bmjopen-2021-053753)
Supplement: Supplementary data [file bmjopen-2021-053753supp001.pdf]

Supplementary Table 1 Maternal characteristics by maternal ethnicity

	White British (n=142)	Pakistani (n=324)
**GDM treatment**, n (%)		
Lifestyle changes advice alone	37 (26.1)	111 (34.3)
Pharmaceutical treatment	105 (73.9)	213 (65.7)
**Age at childbirth (years)**, median (IQR)	30.0 (9.4)	30.8 (7.4)
**BMI at booking (kg/m^2)**, median (IQR)	27.9 (10.5)	26.9 (7.1)
**BMI category at booking** , n (%)		
Underweight *(BMI< 18.5 kg/m^2)*	2 (1.4)	7 (2.2)
Normal weight *(18.5≤BMI≤24.9 kg/m^2)*	53 (37.3)	99 (30.6)
Overweight *(25.0≤BMI≤29.9 kg/m^2)*	29 (20.4)	121 (37.3)
Obese *(BMI ≥30.0 kg/m^2)*	58 (40.8)	97 (29.9)
**Smoking during pregnancy**, n (%)		
Yes	39 (27.5)	10 (3.1)
No	103 (72.5)	314 (96.9)
**Parity,** n (%)		
0	68 (47.9)	94 (29.0)
1	43 (30.3)	63 (19.4)
2	20 (14.1)	62 (19.1)
3+	11 (7.7)	105 (32.4)
**Physical activity levels,** n (%)		
Inactive	53 (37.3)	254 (78.4)
Moderately inactive	37 (26.1)	41 (12.6)
Moderately active	37 (26.1)	21 (6.5)
Active	15 (10.6)	8 (2.5)
**Highest educational qualification,** n (%)		
5 GCSE equivalent or less	60 (42.2)	192 (59.3)
A-level equivalent	25 (17.6)	30 (9.3)
Higher than A-level	40 (28.2)	85 (26.2)
Other/Unknown	17 (12.0)	17 (5.2)
**Family history of diabetes,** n (%)		
Yes	75 (52.8)	221 (68.2)
No	67 (47.2)	103 (31.8)
**Mother's employment status,** n (%)		
Currently employed	105 (73.9)	65 (20.1)
Previously employed	31 (21.8)	90 (27.8)
Never employed	6 (4.2)	169 (52.2)
**Gestational age at OGTT (weeks),** median (IQR)	26.3 (1.1)	26.3 (1.1)
**Fasting glucose concentrations at OGTT (mmol/L),** median (IQR)	4.7 (0.8)	5.1 (0.9)
**2h post-load glucose concentrations at OGTT (mmol/L),** median (IQR)	8.3 (0.9)	8.6 (1.5)

A-level: UK highest qualification in high school; BMI: body mass index; GCSE: general certificate of secondary education; OGTT: oral glucose tolerance test
Continuous data presented as median and interquartile range (IQR).
Categorical data presented as frequencies and percentages.

Martine-Edith G, *et al. BMJ Open* 2021; 11:e053753. doi: 10.1136/bmjopen-2021-053753

Supplementary Table 2 Associations between maternal characteristics and GDM pharmaceutical treatment relative to lifestyle changes advice stratified by maternal ethnicity

	**White British (N=142)**		**Pakistani (N=324)**	
	**Pharmaceutical treatment**		**Pharmaceutical treatment**	
	Adjusted OR (95% CI)	*p*	*Adjusted OR (95% CI)*	*p*
**Mother age at childbirth (years)**	1.1 (1.0, 1.2)	0.145	1.1 (1.0, 1.2)	0.008
**BMI categories at booking (kg/m^2)**				
Normal weight	***Reference***		***Reference***	
Underweight	1.1 (0.03, 46.8)	0.943	1.2 (0.2, 7.0)	0.798
Overweight	1.0 (0.3, 3.4)	0.954	1.2 (0.6, 2.3)	0.546
Obese	2.1 (0.6, 7.0)	0.215	4.0 (1.8, 8.8)	0.001
**Parity**				
0	***Reference***		***Reference***	
1	0.7 (0.2, 2.3)	0.613	0.5 (0.2, 1.2)	0.157
2	0.6 (0.1, 3.5)	0.611	0.5 (0.2, 1.2)	0.152
3+	0.09 (0.01, 0.8)	0.027	0.6 (0.2, 1.4)	0.208
**Highest educational qualification**				
5 GCSE equivalent or less	***Reference***		***Reference***	
A-level equivalent	0.5 (0.1, 2.1)	0.361	0.8 (0.3, 2.2)	0.678
Higher than A-level	0.7 (0.2, 2.6)	0.577	0.6 (0.3, 1.1)	0.122
Other/Unknown	0.5 (0.1, 2.6)	0.417	1.1 (0.3, 3.8)	0.925
**Employment status**				
Currently employed	***Reference***		***Reference***	
Previously employed	0.5 (0.1, 2.0)	0.328	1.1 (0.4, 2.6)	0.862
Never employed	0.3 (0.02, 3.7)	0.343	0.6 (0.2, 1.3)	0.190
**Physical activity levels**				
Active	***Reference***		***Reference***	
Moderately active	1.3 (0.3, 5.7)	0.737	0.3 (0.05, 2.3)	0.281
Moderately inactive	1.7 (0.4, 7.5)	0.502	1.1 (0.2, 6.6)	0.912
Inactive	3.1 (0.7, 13.5)	0.134	0.9 (0.1, 4.9)	0.883
**Smoking during pregnancy**	2.1 (0.6, 7.4)	0.247	3.5 (0.4, 33.6)	0.269
**Family history of diabetes**	2.5 (1.0, 6.7)	0.058	1.0 (0.5, 1.8)	>0.999
**Gestational age at OGTT (weeks)**	0.9 (0.7, 1.2)	0.605	0.9 (0.8, 1.0)	0.070
**Fasting glucose at OGTT (mmol/L)**	2.5 (0.9, 6.5)	0.066	1.7 (1.1, 2.4)	0.008
**2h post-load glucose at OGTT (mmol/L)**	2.3 (1.0, 5.3)	0.039	1.2 (1.0, 1.5)	0.049

BMI: body mass index; CI: confidence interval; OGTT: oral glucose tolerance test

Martine-Edith G, *et al. BMJ Open* 2021; 11:e053753. doi: 10.1136/bmjopen-2021-053753
