# Supplementary material for: Associations between maternal characteristics and pharmaceutical treatment of gestational diabetes: an analysis of the UK Born in Bradford (BiB) cohort study
Source: BMJ Open. 2021 Nov 3;11(11):e053753. doi: 10.1136/bmjopen-2021-053753 (PMC8572403; doi:10.1136/bmjopen-2021-053753)
Supplement: Author's manuscript [file bmjopen-2021-053753.draft_revisions.pdf]

BMJ Open is committed to open peer review. As part of this commitment we make the peer review history of every article we publish publicly available.

When an article is published we post the peer reviewers' comments and the authors' responses online. We also post the versions of the paper that were used during peer review. These are the versions that the peer review comments apply to.

The versions of the paper that follow are the versions that were submitted during the peer review process. They are not the versions of record or the final published versions. They should not be cited or distributed as the published version of this manuscript.

BMJ Open is an open access journal and the full, final, typeset and author-corrected version of record of the manuscript is available on our site with no access controls, subscription charges or pay-per-view fees (http://bmjopen.bmj.com).

If you have any questions on BMJ Open's open peer review process please email info.bmjopen@bmj.com

BMJ Open

Pharmacological management of gestational diabetes and the introduction of metformin: an analysis of the UK Born in Bradford (BiB) cohort study

Journal:	*BMJ Open*
Manuscript ID	bmjopen-2021-053753
Article Type:	Original research
Date Submitted by the Author:	24-May-2021

Complete List of Authors:	Martine-Edith, Gilberte; Loughborough University,<br>Johnson, William; Loughborough University<br>Hunsicker, Eugenie; Loughborough University<br>Hamer, Mark; University College London<br>Petherick, Emily; Loughborough University

Keywords:	Diabetes in pregnancy < DIABETES & ENDOCRINOLOGY, EPIDEMIOLOGY, PUBLIC HEALTH

SCHOLARONE™
Manuscripts

BMJ

I, the Submitting Author has the right to grant and does grant on behalf of all authors of the Work (as defined in the below author licence), an exclusive licence and/or a non-exclusive licence for contributions from authors who are: i) UK Crown employees; ii) where BMJ has agreed a CC-BY licence shall apply, and/or iii) in accordance with the terms applicable for US Federal Government officers or employees acting as part of their official duties; on a worldwide, perpetual, irrevocable, royalty-free basis to BMJ Publishing Group Ltd ("BMJ") its licensees and where the relevant Journal is co-owned by BMJ to the co-owners of the Journal, to publish the Work in this journal and any other BMJ products and to exploit all rights, as set out in our licence.

The Submitting Author accepts and understands that any supply made under these terms is made by BMJ to the Submitting Author unless you are acting as an employee on behalf of your employer or a postgraduate student of an affiliated institution which is paying any applicable article publishing charge ("APC") for Open Access articles. Where the Submitting Author wishes to make the Work available on an Open Access basis (and intends to pay the relevant APC), the terms of reuse of such Open Access shall be governed by a Creative Commons licence – details of these licences and which Creative Commons licence will apply to this Work are set out in our licence referred to above.

Other than as permitted in any relevant BMJ Author's Self Archiving Policies, I confirm this Work has not been accepted for publication elsewhere, is not being considered for publication elsewhere and does not duplicate material already published. I confirm all authors consent to publication of this Work and authorise the granting of this licence.

For peer review only

Pharmacological management of gestational diabetes and the introduction of metformin: an analysis of the UK Born in Bradford (BiB) cohort study

Gilberte Martine-Edith[1], MSc, William Johnson[1], PhD, Eugenie Hunsicker[2], PhD, Mark Hamer[3], PhD, and Emily S Petherick[1], PhD

[1]School of Sport, Exercise and Health Sciences, Loughborough University, Loughborough, UK

[2]School of Science, Loughborough University, Loughborough, UK

[3]Institute of Sport, Exercise and Health, Division Surgery Interventional Science, University College London, London, UK

Corresponding author: William Johnson, School of Sport, Exercise and Health Sciences, Loughborough University, Epinal Way, LE11 3TU, Loughborough, UK. E-mail: w.o.johnson@lboro.ac.uk

For peer review only

For peer review only - http://bmjopen.bmj.com/site/about/guidelines.xhtml

Abstract

Objectives To identify maternal characteristics associated with pharmacological treatment of gestational diabetes mellitus (GDM) and explore the effects of metformin introduction.

Methods We investigated maternal records from 762 Born in Bradford cohort participants receiving GDM treatment. Univariate associations between maternal characteristics and GDM treatment (lifestyle changes vs pharmacological treatment) were examined using Mann-Whitney and Chi-square tests. Receiver operating curve analysis compared the prediction of pharmacological treatment between a minimal model (significant variables from univariate analysis) and a full model (variables selected through multivariable LASSO regression). In the period after metformin introduction, univariate associations between maternal characteristics and GDM treatment (lifestyle changes vs insulin vs metformin) were explored using Kruskal-Wallis and Chi-square tests.

Results Women prescribed pharmacological treatment were older (median: 31.7 years (interquartile range, IQR:7.6)), more hyperglycaemic, and had higher median BMI (28.4 (IQR:7.9)). LASSO-selected variables led to a 2.7% sensitivity and 4.3% specificity improvement in the prediction of pharmacological treatment. After metformin introduction, insulin was prescribed to the most hyperglycaemic women whilst metformin was prescribed to women with high BMI.

Conclusions Higher age, glucose levels and BMI were characteristic of GDM pharmacological management. Metformin introduction decreased insulin prescriptions for mothers with high BMI.

For peer review only

Strengths and limitations of this study

- This study was based on a large sample of women diagnosed with GDM in a centre where universal GDM screening is in place.
- This study allowed for an exploration of the changes in GDM pharmacological treatment following metformin introduction.
- Our results may be biased by clinicians' preference for a specific treatment and patient compliance to treatment.

For peer review only

For peer review only - http://bmjopen.bmj.com/site/about/guidelines.xhtml

Introduction

Gestational diabetes mellitus (GDM) is one of the most common complications of pregnancy [1]. In 2019, the International Diabetes Federation estimated that 13.2% of pregnancies, or 17 million live births, were affected by GDM worldwide [2]. The reported prevalence of GDM is 5% in the United Kingdom (UK) [3]. The public health significance of GDM lies in the intergenerational cycle of diabetes and obesity risk it perpetuates as GDM is associated with both maternal complications (e.g. pre-eclampsia, caesarean delivery) and health risks for the offspring (e.g. macrosomia, childhood obesity) [4].

Guidelines for initial GDM management recommend lifestyle changes (dietary and exercise advice) [5,6]. While these changes are largely effective, hyperglycaemia persists for 15-30% of women and supplemental pharmacological treatment is required [5]. Historically, subcutaneous insulin was the first-line pharmacological agent [5]. However, metformin has been increasingly accepted following the Metformin in Gestational diabetes (MiG) trial that validated it as a safe alternative to insulin [7], despite uncertainties regarding its long-term effects on offspring health [8]. In the UK, both the 2008 and 2015 National Institute for Health and Care Excellence (NICE) guidelines initially recommend metformin for GDM treatment and insulin is suggested when metformin is contraindicated, not tolerated or ineffective [6].

With the aim to inform clinical management of GDM, previous research has investigated the characteristics associated with failure of lifestyle changes to achieve euglycaemia and the subsequent need for supplemental pharmacological treatment in mothers with GDM [9–15]. High maternal body mass index (BMI), history of GDM, advanced age and adverse oral

For peer review only

glucose tolerance test (OGTT) were amongst factors increasing the probability of receiving pharmacological treatment. However, most previous studies exclusively compared insulin to lifestyle changes treatment [9–13] and with the limited number of UK studies[14–16], both the effects of metformin introduction on GDM management and the characteristics of pharmacologically treated women in the UK remain largely unknown.

Using a UK birth cohort that included women with GDM treated both before and after metformin introduction, this study aimed to (1) for the overall study period, identify the maternal characteristics associated with GDM pharmacological treatment, (2) after metformin introduction, compare maternal characteristics between lifestyle changes, insulin and metformin treatment groups.

For peer review only

Methods

Study

Born in Bradford (BiB) is a longitudinal prospective birth cohort study [17]. Bradford, a city in the north of England, constitutes a multi-ethnic population of more than 500,000 individuals, with 20% of the population of South Asian origin. Data were collected between 2007 and 2010 from 12,453 women (and their partners and offspring) booked for delivery at the Bradford Royal Infirmary [18]. Ethical approval for the study was granted by Bradford Research Ethics Committee (Ref 07/H1302/112).

For peer review only - http://bmjopen.bmj.com/site/about/guidelines.xhtml

Patient and public involvement

Patients and/or the public were not involved in the design, conduct, reporting or disseminations plans of this research.

Sample

Our sample comprised 762 women with data on maternal characteristics (Figure 1). Cohort participants diagnosed with GDM in a singleton pregnancy were included if they received (i) lifestyle changes advice only, (ii) lifestyle changes advice with supplementary insulin or (iii) lifestyle changes advice supplemented by metformin. We excluded GDM treatment combinations (e.g. lifestyle changes advice supplemented by both metformin and insulin treatment) that did not yield sufficient numbers for meaningful analyses to be conducted. Participants with GDM for whom treatment was not recorded were excluded. If mothers had more than one singleton pregnancy affected and treated for GDM during the study, we only included the first pregnancy. Singleton pregnancies not affected by GDM and higher order pregnancies (twins, triplets) whether or not affected by GDM were excluded from the study, as were women with pre-existing diabetes.

Screening and diagnosis of GDM

All women enrolled in the BiB study were offered GDM screening. This was conducted between 26 and 28 weeks of gestation using the 2-hour 75g OGTT and 80% of women attended their appointment [18]. Diagnosis of GDM was made using the modified 1999 World Health Organisation (WHO) criteria in accordance with local recommendations at the

For peer review only - http://bmjopen.bmj.com/site/about/guidelines.xhtml

time of recruitment (fasting glucose concentration ≥6.1mmol/L and/or 2-hour post-load glucose ≥7.8mmol/L) [19].

Management and treatment of GDM

Local procedure meant that all women were referred to the joint obstetric diabetes clinic following a diagnosis of GDM. Women were educated in dietary and exercise changes and capillary glucose monitoring. Individualized dietary recommendations were provided by a dietician and daily walking for at least 30 minutes was recommended. If glucose targets were achieved after a week (fasting plasma glucose: 4.0-5.5mmol/L; 2-hour postprandial: ≤7.5mmol/L), lifestyle changes were continued without additional pharmacological treatment. If hyperglycaemia persisted, treatment was supplemented with insulin injections until delivery in the first part of the study (04/2007-03/2009). Following metformin introduction (04/2009), both insulin injections and metformin tablets (850 mg, twice daily) were pharmacological prescription options.

Study outcome: GDM treatment type

The three reported treatment options evaluated in our study were: counselling for lifestyle changes, insulin and metformin. Lifestyle changes consisted of diet and exercise. Insulin and metformin groups included women who initially received lifestyle changes advice followed by supplementary insulin and metformin treatment, respectively.

For peer review only

For peer review only - http://bmjopen.bmj.com/site/about/guidelines.xhtml

Maternal characteristics

Socio-demographic characteristics

Seven socio-demographic characteristics were considered: age at childbirth, marital and cohabitation status, ethnicity (White British, Pakistani, other), employment status (previously, currently, or never employed), migration status, educational levels, and parity. These were self-reported using interviewer-administered questionnaires at booking conducted in English or South Asian languages (e.g. Bengali, Punjabi). Ethnicity was grouped according to the UK Office of National Statistics guidelines [20]. Education levels corresponded to ≤five General Certificate of Secondary Education (GCSE) qualification, A level equivalent, higher than A level and other/unknown. Migration status was classified in two groups: mother was born in the UK or moved to the UK at ≤five years old and mother moved to the UK >five years of age. Marital and cohabitation status was defined as married and living with a partner, not married and living with a partner or not living with a partner.

Lifestyle and health characteristics

Nine lifestyle and health variables were analysed: BMI at booking, smoking during pregnancy (yes/no), physical activity levels, family history of diabetes (yes/no), history of GDM before the study (yes/no), pre-existing hypertension (yes/no), gestational age and blood glucose concentrations at OGTT (fasting and 2-hour post-load) and start date of treatment relative to metformin introduction (before/after). Maternal BMI was obtained from height and weight measurements conducted at recruitment using Leicester Height Measure and SECA digital scales. Family history of diabetes, history of GDM and pre-existing hypertension were self-reported. Gestational age was recorded, and plasma glucose levels were measured at OGTT using a glucose oxidase method. Maternal physical activity levels (inactive,

For peer review only - http://bmjopen.bmj.com/site/about/guidelines.xhtml

moderately inactive, moderately active, active) were self-reported using the UK General Practice Physical Activity Questionnaire [21].

Statistical analysis

Analyses were based on two time periods to account for the fact that metformin was used for GDM treatment in the study from April 2009 onwards.

Overall study period: 04/2007-02/2011

Using the whole study sample, we considered two treatment types: lifestyle changes and pharmacological treatment (i.e. insulin- and metformin-treated women were grouped). Firstly, univariate associations between maternal characteristics and GDM treatment type were explored using the Mann-Whitney U test for continuous variables and Chi-square (or Fisher's exact) test for categorical variables. The Holm-Bonferroni correction adjusted for multiple testing. Secondly, multivariable models were developed. A least absolute shrinkage and selection operator (LASSO) binary regression model was fitted including all maternal characteristics. The characteristics most predictive of pharmacological treatment were selected by LASSO using a 10-fold cross-validation [22].

We then compared two multivariable models. The minimal model included the variables that have shown significance (accounting for multiple testing) following the univariate analysis described above. The full model comprised the LASSO-selected variables. We evaluated if the full model would improve the prediction of pharmacological treatment using a receiver operating characteristics (ROC) curve analysis. Area under the ROC curve (AUC),

For peer review only - http://bmjopen.bmj.com/site/about/guidelines.xhtml

specificity, sensitivity, positive predictive value (PPV) and negative predictive value (NPV) were calculated. The optimal cut-off point was defined as the point of maximum sensitivity and specificity, where the ROC curve was closest to the upper left corner. Additionally, the fit of the two models was evaluated using a Chi-square difference test.

Period after metformin introduction: 04/2009-02/2011

Using the subsample of women who started GDM treatment after metformin introduction, we considered three treatment types: lifestyle changes, insulin, and metformin. Univariate associations between LASSO-selected maternal characteristics and GDM treatment type were examined. The Kruskal-Wallis test was used for continuous variables and the Chi-square (or Fisher's exact) test was used for categorical variables. The Holm-Bonferroni correction adjusted for multiple testing. Further subsample analysis assessed if metformin introduction was associated with changes in the maternal characteristics of women receiving insulin.

Analyses were conducted using R (R 3.4.1 & R Studio 1.0.153 for Windows) and Stata/SE software (Stata/SE 15 for Windows; StataCorp, College Station, TX, USA).

Results

A total of 844 women were diagnosed with GDM in a singleton pregnancy. We excluded 82 women who did not meet our treatment inclusion criteria, leading to a sample of 762 women (Figure 1).

For peer review only

Overall study period: lifestyle changes vs pharmacological treatment

32% of women received lifestyle changes advice alone and 68% received supplemental pharmacological treatment during the study (Table 1). Compared to women receiving lifestyle changes advice, women who had pharmacological treatment were older at childbirth (median age: 31.7 years (interquartile range, IQR: 7.6) vs 29.9 years (8.1)), more hyperglycaemic at OGTT and had higher obesity rates (41.7% vs 19.0%). Mothers in the pharmacological treatment group were also more likely to be smokers, have a history of GDM and family history of diabetes. Differences in age, BMI and glucose concentrations at OGTT between lifestyle changes and pharmacological treatment groups remained significant after accounting for multiple testing (Table 1).

The first variables selected through LASSO regression analysis were BMI at booking, glucose concentrations at OGTT and age at childbirth (Supplementary Table 1). These selected variables were the same as the maternal characteristics shown, in the univariate analyses, to be significantly associated with pharmacological treatment after adjusting for multiple testing (as described above). LASSO also selected smoking, physical activity, family history of diabetes, gestational age at OGTT, employment status, ethnicity, parity and education as predictors of pharmacological treatment in women with GDM (Supplementary Table 1).

ROC analysis showed that the AUC of the full model including the LASSO-selected characteristics (0.8) was greater than the AUC of the minimal model including age, BMI and glucose concentrations at OGTT (0.7). At the optimal cut-off point, the full model was associated with a 2.7% sensitivity improvement (72.4% vs 69.7% in the minimal model) and

For peer review only - http://bmjopen.bmj.com/site/about/guidelines.xhtml

a 4.3% specificity improvement (69.2% vs 64.9%). PPV and NPV were higher for the full model (PPV: 82.5%; NPV: 55.6%) than the minimal model (PPV: 79.9%; NPV: 51.7%). The significance of the Chi-square difference test (p=0.004) confirmed that the variables included in the full model, rather than the minimal model, provided a better fit for pharmacological treatment prediction.

Period after metformin introduction: lifestyle changes vs insulin vs metformin treatment

After metformin introduction, 396 women received GDM treatment (Table 2). Of these, 31.1%, 50.5% and 18.4% respectively received lifestyle changes advice, insulin and metformin treatment. Mothers in the lifestyle changes group were more likely to be younger, less hyperglycaemic and have a lower BMI. Women who were prescribed metformin had the highest median BMI (29.3 (IQR:6.5)) while insulin prescription was mostly provided to older mothers (highest median age: 31.8 years (IQR:8.2)) and those more hyperglycaemic at OGTT (Table 2).

After metformin introduction, there was a decrease in the proportion of insulin-treated overweight (before:69.9%; after:45.1%) and obese women (before:77.5%; after:61.6%) (Supplementary Table 2). The rates of overweight and obese women treated with metformin reached 19.5% and 24.8%, respectively. Age at childbirth and median glucose concentrations at OGTT of insulin-treated mothers remained relatively unchanged following metformin introduction (Supplementary Table 2).

For peer review only - http://bmjopen.bmj.com/site/about/guidelines.xhtml

For peer review only

Discussion

Our study showed that pharmacologically treated women with GDM were older, more obese and hyperglycaemic than mothers receiving lifestyle changes advice. Factors such as ethnicity, smoking and parity may also be associated with pharmacological treatment. Metformin introduction did not, at the time of the study, radically change GDM pharmacological management as most women were still prescribed insulin. However, after metformin introduction, women with high BMI formerly treated with insulin were subsequently treated with metformin while the most hyperglycaemic women were consistently prescribed insulin.

Supplemental pharmacological treatment was the most common form of GDM management in our study. This contrasted with previous studies in which mothers with GDM were more frequently managed with lifestyle changes advice [9,11–14,23–25]. These disparities could be due to differences in GDM diagnostic criteria: the modified 1999 WHO criteria in our study used higher fasting glucose thresholds at OGTT but lower 2-hour thresholds than other criteria in by previous studies [9,13,25]. We hypothesise that the larger representation of South Asian women (notably Pakistani (59%)) in our study compared to previous work [9,14] also contributed to the higher pharmacological treatment rates in our sample as Pakistani women are generally more likely to develop GDM and prone to more severe hyperglycaemia [26]. Finally, the high levels of deprivation in Bradford and the presence of non-English-speaking communities [18] could have limited health literacy and the adherence to lifestyle changes advice [27] in our sample, leading to a greater pharmacological treatment risk.

For peer review only - http://bmjopen.bmj.com/site/about/guidelines.xhtml

Advanced maternal age, obesity and severe hyperglycaemia were the most important characteristics associated with pharmacological treatment of any kind, as shown by both univariate and multivariable analyses. Our results were in line with previous evidence, including from large international population studies [9,12,13], showing that pharmacologically treated women tend to have poorer health characteristics than women receiving lifestyle changes advice alone [9,11–13,23–25]. For instance, the study by Zhang *et al*. [13] highlighted that clinical factors such as higher glucose levels at OGTT increased the risk of insulin treatment whilst Gonzalez-Quintero *et al*. [12] demonstrated that pre-gestational obesity and history of GDM, amongst other factors, were determinant of insulin requirement. Thus, despite the specifics of the BiB cohort described previously, we have shown that pharmacologically treated mothers in our sample and previous studies shared similar key characteristics. It seems that regardless of study location, screening methods and diagnostic criteria, there is a group of women with GDM with one or a combination of clinical risk factors that increase their likelihood of requiring pharmacological intervention to achieve euglycaemia.

Additional socio-demographic and lifestyle characteristics such as smoking, ethnicity and parity improved model fit for the prediction of pharmacological treatment as demonstrated by the ROC analysis. This suggests that, in addition to age, BMI and glycaemic levels, other maternal factors significantly contributed to the risk of receiving pharmacological treatment in our study. In previous literature, smoking was more common among insulin-treated women than women treated with lifestyle changes but no significant differences have been found [12,23,24]. Further, some studies found that pharmacologically treated women were more likely to be multiparous than women receiving lifestyle changes [9,11,12,23], however others did not demonstrate significant differences in parity [24]. There is no consensus

For peer review only - http://bmjopen.bmj.com/site/about/guidelines.xhtml

regarding ethnicity as different ethnic groups have been shown to be most at risk of insulin treatment: Middle Eastern/North African [23], Middle Eastern [9] and Anglo-European [10]. More research is required to gain a greater understanding of the relationships between socio-demographic and lifestyle factors and GDM pharmacological treatment. This could supplement the approach to GDM management beyond the standard clinical risk factors.

The addition of metformin to the set of pharmacological options was not associated, at the time of the study, with any substantial shift in GDM management as insulin remained the most common prescribed treatment. Nevertheless, we have found that, after metformin introduction, metformin-treated women had the highest BMI which is in line with a study by McGrath *et al.* [28]. However, Ijas *et al.* [29] compared metformin only to metformin plus insulin groups and demonstrated that metformin was more likely to be effective for women with lower BMI. In our study however, the decrease in the proportion of insulin treatment for overweight and obese women after metformin introduction suggests that metformin may have been used as an alternative to insulin for mothers with the highest BMI. Further, we found that women with more severe hyperglycaemia were consistently prescribed insulin rather than metformin, which corroborated previous research [28–30]. As metformin is believed to act less rapidly than insulin [15], it may be that in our study, even after metformin introduction, the most hyperglycaemic women were preferentially prescribed insulin to promptly restore normoglycaemia. Finally, the differences in maternal characteristics between pharmacological treatment types may also be explained by additional non-clinical factors that could have varying degrees of impact on the decision to treat GDM with insulin or metformin. These include the cost implications of treatment (metformin tends to be less expensive than insulin) [31], maternal treatment preference (e.g. preference for metformin as insulin injections are invasive) and cultural barriers to compliance [27].

For peer review only

The main strength of this study is that our findings are based on a large sample of women diagnosed with GDM from a cohort where universal GDM screening was in place. Further, all data originated from a single diabetes clinic in the UK managed by the same clinician and where the same diagnostic criteria and glucose targets for GDM management were used throughout the study. Although this may limit the generalisability of our results, this also minimised bias related to differences in clinical practice and decisions between clinics. We could not however control for clinicians' preference for a specific treatment or patient compliance to treatment. Another strength of our study is that, unlike previous studies that explored maternal characteristics of GDM treatment either before or after metformin introduction, our data captured GDM management both pre- and post-metformin introduction. This allowed for an analysis of the changes in GDM pharmacological management when metformin was first introduced, which, to the best of our knowledge, has never been conducted before. We are however limited by the relatively small sample of women treated with metformin at the time of the BiB study. We expect that our study could be reproduced using data from an updated BiB cohort, to examine the differences in characteristics between insulin- and metformin-treated mothers now that metformin has been used for more than a decade.

To conclude, in the UK BiB cohort, consistently with previous research, women with GDM who were older, more hyperglycaemic and had higher BMI were more likely to require pharmacological treatment. When metformin was first introduced as GDM treatment, it led to changes in GDM management according to maternal weight status but not glycaemic status.

For peer review only - http://bmjopen.bmj.com/site/about/guidelines.xhtml

Further research in the UK is needed to determine to which extent the maternal determinants of GDM pharmacological treatment impact on obstetric and neonatal outcomes.

For peer review only

For peer review only - http://bmjopen.bmj.com/site/about/guidelines.xhtml

Acknowledgements

Born in Bradford is only possible because of the enthusiasm and commitment of the children and parents in BiB. We are grateful to all the participants, health professionals, schools and researchers who have made Born in Bradford happen.

Declarations

Funding

This research was funded by Loughborough University and supported by the National Institute for Health Research (NIHR) Leicester Biomedical Research Centre. E.S.P. and W.J. acknowledge support from the National Institute for Health Research (NIHR) Leicester Biomedical Research Centre, which is a partnership between University Hospitals of Leicester NHS Trust, Loughborough University, and the University of Leicester. W.J. is supported by a UK Medical Research Council (MRC) New Investigator Research Grant (MR/P023347/1). Born in Bradford received funding from a Wellcome Trust infrastructure grant (WT101597MA), the National Institute for Health Research under its Collaboration for Applied Health Research and Care (CLAHRC) (IS-CLA-0113-10020). The NIHR Clinical Research Network which provided research delivery support for this study.

The views expressed in this paper are those of the authors and not necessarily those of the NIHR.

Authors' contribution

G.M-E. wrote the manuscript and was responsible for the acquisition, analysis, and interpretation of data. E.S.P. and W.J. revised the manuscript and contributed to the

For peer review only - http://bmjopen.bmj.com/site/about/guidelines.xhtml

acquisition, analysis, and interpretation of data. E.H. contributed to the analysis and interpretation of data and reviewed the manuscript. M.H. reviewed the manuscript. G.M-E., W.J. and E.S.P. are guarantors of this work and, as such, had full access to all the data in the study and take responsibility for the integrity of the data and the accuracy of the data analysis.

Declarations of interest

None

Data availability

Scientists are encouraged and able to use BiB data. Data requests are made to the BiB executive using the form available from the study website http://www.borninbradford.nhs.uk. Guidance for researchers and collaborators, the study protocol and the data collection schedule are all available via the website. All requests are carefully considered and accepted where possible.

Ethics approval

Ethical approval for the study was granted by Bradford Research Ethics Committee (Ref 07/H1302/112).

Informed consent

All participants provided written consent for the BiB study.

For peer review only - http://bmjopen.bmj.com/site/about/guidelines.xhtml

For peer review only

References

1 American Diabetes Association. Diagnosis and Classification of Diabetes Mellitus. *Diabetes Care* 2008;**31**:S55–60. doi:10.2337/dc08-S055

2 International Diabetes Federation. *IDF Diabetes Atlas 2019*. 2019. http://www.idf.org/about-diabetes/facts-figures

3 Diabetes UK. Us, diabetes and a lot of facts and stats. London: 2019. www.diabetes.org.uk

4 Chen L, Mayo R, Chatry A, *et al.* Gestational Diabetes Mellitus: Its Epidemiology and Implication beyond Pregnancy. *Curr Epidemiol Reports* 2016;**3**:1–11. doi:10.1007/s40471-016-0063-y

5 American Diabetes Association. Management of diabetes in pregnancy: Standards of medical care in diabetes. *Diabetes Care* 2019;**42**:S165–72. doi:10.2337/dc19-S014

6 National Institute for Health and Care. Diabetes in pregnancy : management from preconception to the postnatal period. 2015.

7 Rowan JA, Hague WM, Gao W, *et al.* Metformin versus Insulin for the Treatment of Gestational Diabetes. *N Engl J Med* 2008;**358**:2003–15. doi:10.1056/NEJMoa0707193

8 Lindsay RS, Loeken MR. Metformin use in pregnancy: promises and uncertainties. *Diabetologia* 2017;**60**:1612–9. doi:10.1007/s00125-017-4351-y

9 Barnes RA, Wong T, Ross GP, *et al.* A novel validated model for the prediction of insulin therapy initiation and adverse perinatal outcomes in women with gestational diabetes mellitus. *Diabetologia* 2016;**59**:2331–8. doi:10.1007/s00125-016-4047-8

10 Wong VW, Jalaludin B. Gestational diabetes mellitus: Who requires insulin therapy? *Aust New Zeal J Obstet Gynaecol* 2011;**51**:432–6. doi:10.1111/j.1479-828X.2011.01329.x

11 Aktun LH, Yorgunlar B, Karaca N, *et al.* Predictive Risk Factors in the Treatment of Gestational Diabetes Mellitus. *Clin Med Insights Women's Heal* 2015;**8**:CMWH.S31564. doi:10.4137/cmwh.s31564

12 González-Quintero VH, Istwan NB, Rhea DJ, *et al.* Antenatal Factors Predicting Subsequent Need for Insulin Treatment in Women with Gestational Diabetes. *J Women's Heal* 2008;**17**:1183–7. doi:10.1089/jwh.2007.0667

13 Zhang Y, Shao J, Li F, *et al.* Factors in Gestational Diabetes Mellitus Predicting the Needs for Insulin Therapy. *Int J Endocrinol* 2016;**2016**:1–5. doi:10.1155/2016/4858976

14 Ali A, Shastry S, Nithiyananthan R, *et al.* Gestational diabetes–Predictors of response to treatment and obstetric outcome. *Eur J Obstet Gynecol Reprod Biol* 2018;**220**:57–60. doi:10.1016/j.ejogrb.2017.11.014

15 Khin MO, Gates S, Saravanan P. Predictors of metformin failure in gestational diabetes mellitus (GDM). *Diabetes Metab Syndr Clin Res Rev* 2018;**12**:405–10. doi:10.1016/j.dsx.2018.01.003

For peer review only - http://bmjopen.bmj.com/site/about/guidelines.xhtml

16 Gandhi P, Bustani R, Madhuvrata P, *et al.* Introduction of metformin for gestational diabetes mellitus in clinical practice: Has it had an impact? *Eur J Obstet Gynecol Reprod Biol* 2012;**160**:147–50. doi:10.1016/j.ejogrb.2011.11.018

17 Raynor P, Duley L, Small N, *et al.* Born in Bradford, a cohort study of babies born in Bradford, and their parents: Protocol for the recruitment phase. *BMC Public Health* 2008;**8**:1–13. doi:10.1186/1471-2458-8-327

18 Wright J, Small N, Raynor P, *et al.* Cohort profile: The born in bradford multi-ethnic family cohort study. *Int J Epidemiol* 2013;**42**:978–91. doi:10.1093/ije/dys112

19 World Health Organisation. Definition, diagnosis and classification of diabetes mellitus and its complications. Report of a WHO consultation. Part 1: diagnosis and classification of diabetes mellitus. https://apps.who.int/iris/handle/10665/66040

20 Office for National Statistics. Ethnic group statistics: a guide for the collection and classification of ethnicity data - Office for National Statistics. Off. Natl. Stat. 2003.https://www.ons.gov.uk/methodology/classificationsandstandards/measuringequality/ethnicgroupnationalidentityandreligion

21 National Health Service . The General Practice Physical Activity Questionnaire (GPPAQ): A screening tool to assess adult physical activity levels, within primary care. 2009. doi:10.1007/s00330-011-2164-9

22 Vincent M, Hansen NR. Sparse group lasso and high dimensional multinomial classification. *Comput Stat Data Anal* 2014;**71**:771–86. doi:10.1016/j.csda.2013.06.004

23 Koning SH, Scheuneman KA, Lutgers HL, *et al.* Risk stratification for healthcare planning in women with gestational diabetes mellitus. *Neth J Med* 2016;**74**:262–9.

24 Sapienza AD, Francisco RPV, Trindade TC, *et al.* Factors predicting the need for insulin therapy in patients with gestational diabetes mellitus. *Diabetes Res Clin Pract* 2010;**88**:81–6. doi:10.1016/j.diabres.2009.12.023

25 Nishikawa T, Ono K, Hashimoto S, *et al.* One-hour oral glucose tolerance test plasma glucose at gestational diabetes diagnosis is a common predictor of the need for insulin therapy in pregnancy and postpartum impaired glucose tolerance. *J Diabetes Investig* 2018;**9**:1370–7. doi:10.1111/jdi.12848

26 West J, Lawlor DA, Fairley L, *et al.* Differences in socioeconomic position, lifestyle and health-related pregnancy characteristics between Pakistani and White British women in the Born in Bradford prospective cohort study: The influence of the woman's, her partner's and their parents' place . *BMJ Open* 2014;**4**. doi:10.1136/bmjopen-2014-004805

27 Draffin CR, Alderdice FA, McCance DR, *et al.* Exploring the needs, concerns and knowledge of women diagnosed with gestational diabetes: A qualitative study. *Midwifery* 2016;**40**:141–7. doi:10.1016/j.midw.2016.06.019

28 McGrath R, Glastras S, Scott E, *et al.* Outcomes for Women with Gestational Diabetes Treated with Metformin: A Retrospective, Case-Control Study. *J Clin Med* 2018;**7**:50. doi:10.3390/jcm7030050

29 Ijas H, Vaarasmaki M, Morin-Papunen L, *et al.* Metformin should be considered in the treatment of gestational diabetes: A prospective randomised study. *BJOG An Int J*

For peer review only - http://bmjopen.bmj.com/site/about/guidelines.xhtml

Obstet Gynaecol 2010;**118**:880–5. doi:10.1111/j.1471-0528.2010.02763.x

30 Goh JEL, Sadler L, Rowan J. Metformin for gestational diabetes in routine clinical practice. *Diabet Med* 2011;**28**:1082–7. doi:10.1111/j.1464-5491.2011.03361.x

31 Ainuddin J, Karim N, Hasan AA, *et al.* Metformin versus insulin treatment in gestational diabetes in pregnancy in a developing country. A randomized control trial. *Diabetes Res Clin Pract* 2015;**107**:290–9. doi:10.1016/j.diabres.2014.10.001

For peer review only

For peer review only - http://bmjopen.bmj.com/site/about/guidelines.xhtml

Table 1 Maternal characteristics by GDM treatment type across the whole study period (2007-2011)

		Lifestyle changes (n=244)	Pharmacological treatment (n=518)	*P*	*P**	n (%) Missing
**Start date of treatment‡**	n (%)			0.486	1.000	4 (0.5)
Before metformin introduction (2007-09)		121 (49.6)	241 (47)			
After metformin introduction (2009-11)		123 (50.4)	273 (53)			
**Age at childbirth† (years)**	Median (IQR)	29.9 (8.1)	31.7 (7.6)	<0.001	0.001	0
**BMI at booking† (kg/m²)**	Median (IQR)	25.2 (6.0)	28.4 (7.9)	<0.001	<0.001	46 (6.0)
**BMI category at booking‡**	n (%)			<0.001	<0.001	46 (6.0)
Underweight *(BMI<18.5 kg/m²)*		7 (3.0)	7 (1.4)			
Normal weight *(18.5≤BMI≤24.9 kg/m²)*		107 (46.1)	122 (25.2)			
Overweight *(25.0≤BMI≤29.9 kg/m²)*		74 (31.9)	153 (31.6)			
Obese *(BMI ≥30.0 kg/m²)*		44 (19.0)	202 (41.7)			
**Smoking during pregnancy‡**	n (%)			0.008	0.096	1 (0.1)
Yes		11 (4.5)	53 (10.2)			
No		233 (95.5)	464 (89.8)			
**Parity‡**	n (%)			0.671	1.000	24 (3.1)
0		88 (37.0)	164 (32.8)			
1		53 (22.3)	119 (23.8)			
2		41 (17.2)	99 (19.8)			
3+		56 (23.5)	118 (23.6)			
**Physical activity levels‡**	n (%)			0.684	1.000	109 (14)
Inactive		134 (62.3)	285 (65.1)			
Moderately inactive		37 (17.2)	77 (17.6)			
Moderately active		35 (16.3)	56 (12.8)			
Active		9 (4.2)	20 (4.6)			
**Ethnic group‡**	n (%)			0.060	0.540	0
White British		47 (19.3)	140 (27.0)			
Pakistani		152 (62.3)	298 (57.5)			
Other		45 (18.4)	80 (15.4)			
**Migration status‡**	n (%)			0.253	1.000	13 (1.7)
Born in the UK or moved ≤ 5 years		121 (51.3)	286 (55.7)			
Moved to the UK > 5 years		115 (48.7)	227 (44.2)			
**Marital and cohabitation status‡**	n (%)			0.239	1.000	0
Married and living with partner		198 (81.1)	411 (79.3)			
Not married and living with partner		21 (8.6)	64 (12.4)			
Not living with partner		25 (10.2)	43 (8.3)			
**Highest educational qualification‡**	n (%)			0.528	1.000	4 (0.5)
5 GCSE equivalent or less		124 (51.0)	273 (53.0)			
A-level equivalent		28 (11.5)	57 (11.1)			
Higher than A-level		76 (31.3)	141 (27.4)			
Other/Unknown		15 (6.2)	44 (8.5)			
**Family history of diabetes‡**	n (%)			0.010	0.100	63 (8.3)
Yes		128 (57.7)	323 (67.7)			
No		94 (42.3)	154 (32.3)			
**Pre-existing hypertension§**	n (%)			0.563	1.000	53 (7.0)
Yes		3 (1.3)	10 (2.1)			
No		228 (98.7)	468 (97.9)			
**History of GDM before the study‡**	n (%)			0.075	0.600	93 (12)

For peer review only

For peer review only - http://bmjopen.bmj.com/site/about/guidelines.xhtml

Yes		10 (4.6)	38 (8.4)			
No		207 (95.4)	414 (91.6)			
**Mother's employment status‡**	n (%)			0.008	0.096	0
Currently employed		90 (36.9)	203 (39.2)			
Previously employed		55 (22.5)	159 (30.7)			
Never employed		99 (40.6)	156 (30.1)			
**Gestational age at OGTT† (weeks)**	Median (IQR)	26.4 (1.6)	26.3 (0.8)	0.006	0.078	13 (1.7)
**Fasting glucose concentrations at OGTT† (mmol/L)**	Median (IQR)	4.7 (0.7)	5.1 (1.1)	<0.001	<0.001	13 (1.7)
**2h post-load glucose concentrations at OGTT† (mmol/L)**	Median (IQR)	8.2 (0.8)	8.6 (1.6)	<0.001	<0.001	13 (1.7)

A-level: UK highest qualification in high school; BMI: body mass index; GCSE: general certificate of secondary education; OGTT: oral glucose tolerance test

Continuous data presented as median and interquartile range (IQR).

Categorical data presented as frequencies and percentages.

*Adjusted *P*-value after Holm-Bonferroni correction

†Mann-Whitney U test

‡Chi-square test

§Fisher's exact test

For peer review only

Table 2 Maternal characteristics by GDM treatment type after metformin introduction (2009-2011)

		Lifestyle changes (n=123)	Insulin (n=200)	Metformin (n=73)	*P*	*P**	n (%) Missing
**Age at childbirth† (years)**	Median (IQR)	29.1 (7.8)	31.8 (8.2)	30.6 (9.0)	<0.001	0.004	0
**BMI at booking† (kg/m²)**	Median (IQR)	24.7 (4.9)	28.1 (9.2)	29.3 (6.5)	<0.001	0.001	21 (2.8)
**BMI category at booking§**	n (%)				<0.001	<0.001	21 (2.8)
Underweight *(BMI<18.5 kg/m²)*		4 (3.4)	5 (2.7)	0			
Normal weight *(18.5≤BMI≤24.9 kg/m²)*		57 (48.3)	55 (29.3)	16 (23.2)			
Overweight *(25.0≤BMI≤29.9 kg/m²)*		40 (33.9)	51 (27.1)	22 (31.9)			
Obese *(BMI ≥30.0 kg/m²)*		17 (14.4)	77 (41.0)	31 (44.9)			
**Smoking during pregnancy‡**	n (%)				0.004	0.032	0
Yes		4 (3.3)	29 (14.5)	6 (8.2)			
No		119 (96.7)	171 (85.5)	67 (91.8)			
**Parity‡**	n (%)				0.145	0.580	6 (0.8)
0		52 (43.0)	68 (34.5)	22 (30.6)			
1		26 (21.5)	46 (23.3)	21 (29.2)			
2		18 (14.9)	35 (17.8)	19 (26.4)			
3+		25 (20.7)	48 (24.4)	10 (13.9)			
**Physical activity levels‡**	n (%)				0.424	0.630	1 (0.1)
Inactive		71 (57.7)	116 (58.3)	43 (58.9)			
Moderately inactive		23 (18.7)	43 (21.6)	9 (12.3)			
Moderately active		22 (17.9)	30 (15.1)	13 (17.8)			
Active		7 (5.7)	10 (5.0)	8 (11.0)			
**Ethnic group‡**	n (%)				0.210	0.630	0
White British		24 (19.5)	54 (27.0)	15 (20.5)			
Pakistani		74 (60.2)	113 (56.5)	50 (68.5)			
Other		25 (20.3)	33 (16.5)	8 (11.0)			
**Highest educational qualification‡**	n (%)				0.297	0.630	0
5 GCSE equivalent or less		53 (43.1)	104 (52.0)	32 (43.8)			
A-level equivalent		17 (13.8)	27 (13.5)	7 (9.6)			
Higher than A-level		44 (35.8)	55 (27.5)	31 (42.5)			
Other/Unknown		9 (7.3)	14 (7.0)	3 (4.1)			
**Family history of diabetes‡**	n (%)				0.099	0.553	27 (3.5)
Yes		65 (57.0)	130 (69.1)	42 (62.7)			
No		49 (43.0)	58 (30.8)	25 (37.3)			
**Mother's employment status‡**	n (%)				0.079	0.553	0
Currently employed		48 (39.0)	80 (40.0)	28 (38.4)			
Previously employed		19 (15.4)	54 (27.0)	20 (27.4)			
Never employed		56 (45.5)	66 (33.0)	25 (34.2)			
**Gestational age at OGTT† (weeks)**	Median (IQR)	26.3 (1.8)	26.1 (0.8)	26.3 (0.7)	0.087	0.553	8 (1.0)
**Fasting glucose concentrations at OGTT† (mmol/L)**	Median (IQR)	4.7 (0.8)	5.2 (1.2)	4.8 (0.7)	<0.001	0.001	8 (1.0)
**2h post-load glucose concentrations at OGTT† (mmol/L)**	Median (IQR)	8.2 (0.8)	8.6 (1.6)	8.4 (1.3)	<0.001	0.001	8 (1.0)

A-level: UK highest qualification in high school; BMI: body mass index; GCSE: general certificate of secondary education; OGTT: oral glucose tolerance test
Continuous data presented as median and interquartile range (IQR).
Categorical data presented as frequencies and percentages.
*Adjusted *P*-values after Holm-Bonferroni correction
†Kruskal-Wallis test
‡Chi-square test
§Fisher's exact test

For peer review only - http://bmjopen.bmj.com/site/about/guidelines.xhtml

For peer review only

Figure 1 legend: Flowchart of study participation

For peer review only

For peer review only - http://bmjopen.bmj.com/site/about/guidelines.xhtml

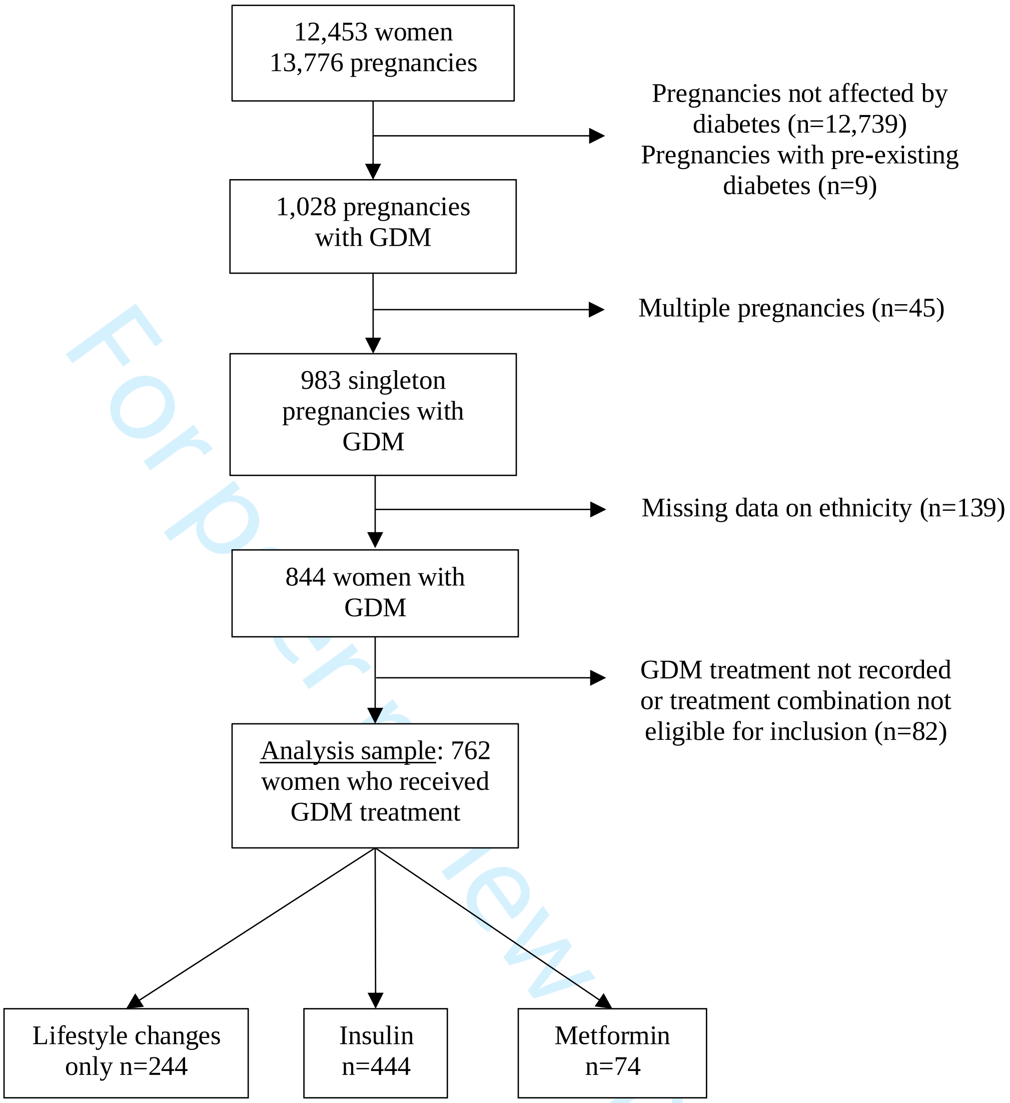

Figure 1 Flowchart of study participation

Supplementary Table 1. Maternal characteristics selected by LASSO: Binary logistic regression estimating coefficients associated with GDM pharmacological treatment

Order of selection	Variable/Level of variable selected	Coefficients
1	BMI at booking (kg/m^2)	0.09
2	Fasting glucose (mmol/L)	0.4
3	Age at childbirth (years)	0.3
4	BMI category: normal weight<br>BMI category: obese	-0.2<br>0.2
5	2-hour post-load glucose (mmol/L)<br>Gestational age at OGTT (weeks)	0.2<br>-0.2
6	Ethnicity: White British	0.2
7	Smoking during pregnancy	0.1
8	Employment status: never employed	-0.05
9	Parity: 3+ children	-0.1
10	Physical activity levels: active	0.03
11	Family history of diabetes<br>Education levels: 5 GCSE equivalent<br>Physical activity levels: moderately active	0.04<br>0.06<br>-0.03
12	Ethnicity: Other	-0.02
13	Employment status: previously employed	0.004
14	Parity: nulliparous	0.0003

BMI: body mass index; GCSE: general certificate of secondary education (UK standard minimum level of education); OGTT: oral glucose tolerance test

For peer review only - http://bmjopen.bmj.com/site/about/guidelines.xhtml

Supplementary Table 2. Maternal characteristics by treatment type before and after metformin

		Before metformin introduction (n=362)		After metformin introduction (n=396)		
		Lifestyle changes (n=121)	Insulin (n=241)	Lifestyle changes (n=123)	Insulin (n=200)	Metformin (n=73)
**Age at childbirth (years)**	Median (IQR)	30.4 (8.0)	31.8 (6.9)	29.1 (7.8)	31.8 (8.2)	30.6 (9.0)
**BMI at booking (kg/m^2)**	Median (IQR)	25.8 (7.0)	28.3 (7.3)	24.7 (4.9)	28.1 (9.2)	29.3 (6.5)
**BMI category at booking**	n (%)					
Underweight *(BMI<18.5 kg/m^2)*		<5	<5	<5	<5	<5
Normal weight *(18.5≤BMI≤24.9 kg/m^2)*		50 (50.0)	50 (50.0)	57 (44.5)	55 (43.0)	16 (12.5)
Overweight *(25≤BMI≤29.99 kg/m^2)*		34 (30.1)	79 (69.9)	40 (35.4)	51 (45.1)	22 (19.5)
Obese *(BMI ≥30 kg/m^2)*		27 (22.5)	93 (77.5)	17 (13.6)	77 (61.6)	31 (24.8)
**Smoking during pregnancy**	n (%)					
Yes		7 (29.2)	17 (70.8)	<5	29 (74.4)	6 (15.4)
No		114 (33.8)	223 (66.2)	119 (33.3)	171 (47.9)	67 (18.8)
**Fasting glucose concentrations at OGTT (mmol/L)**	Median (IQR)	4.7 (0.9)	5.1 (1.3)	4.7 (0.8)	5.2 (1.2)	4.8 (0.7)
**2h post-load glucose concentrations at OGTT (mmol/L)**	Median (IQR)	8.2 (0.9)	8.7 (1.6)	8.2 (0.8)	8.6 (1.6)	8.4 (1.3)

Continuous data presented as median and interquartile range (IQR).
Categorical data presented as frequencies and percentages.
BMI: body mass index; OGTT: oral glucose tolerance test

For peer review only - http://bmjopen.bmj.com/site/about/guidelines.xhtml

STROBE Statement—Checklist of items that should be included in reports of ***cohort studies***

	Item No	Recommendation	Reported on page #
**Title and abstract**	1	(*a*) Indicate the study's design with a commonly used term in the title or the abstract	1
		(*b*) Provide in the abstract an informative and balanced summary of what was done and what was found	2
**Introduction**			
Background/rationale	2	Explain the scientific background and rationale for the investigation being reported	4-5
Objectives	3	State specific objectives, including any prespecified hypotheses	5
**Methods**			
Study design	4	Present key elements of study design early in the paper	5
Setting	5	Describe the setting, locations, and relevant dates, including periods of recruitment, exposure, follow-up, and data collection	5-6
Participants	6	(*a*) Give the eligibility criteria, and the sources and methods of selection of participants. Describe methods of follow-up	6
		(*b*) For matched studies, give matching criteria and number of exposed and unexposed	NA
Variables	7	Clearly define all outcomes, exposures, predictors, potential confounders, and effect modifiers. Give diagnostic criteria, if applicable	7-8
Data sources/ measurement	8*	For each variable of interest, give sources of data and details of methods of assessment (measurement). Describe comparability of assessment methods if there is more than one group	7-8
Bias	9	Describe any efforts to address potential sources of bias	9
Study size	10	Explain how the study size was arrived at	10
Quantitative variables	11	Explain how quantitative variables were handled in the analyses. If applicable, describe which groupings were chosen and why	9-10
Statistical methods	12	(*a*) Describe all statistical methods, including those used to control for confounding	9-10
		(*b*) Describe any methods used to examine subgroups and interactions	9-10
		(*c*) Explain how missing data were addressed	9-10
		(*d*) If applicable, explain how loss to follow-up was addressed	NA
		(*e*) Describe any sensitivity analyses	NA
**Results**			
Participants	13*	(a) Report numbers of individuals at each stage of study—eg numbers potentially eligible, examined for eligibility, confirmed eligible, included in the study, completing follow-up, and analysed	10
		(b) Give reasons for non-participation at each stage	10
		(c) Consider use of a flow diagram	Fig. 1
Descriptive data	14*	(a) Give characteristics of study participants (eg demographic, clinical, social) and information on exposures and potential confounders	10-11
		(b) Indicate number of participants with missing data for each variable of interest	Table 1, 2
		(c) Summarise follow-up time (eg, average and total amount)	NA
Outcome data	15*	Report numbers of outcome events or summary measures over time	10-11
Main results	16	(*a*) Give unadjusted estimates and, if applicable, confounder-adjusted estimates and their precision (eg, 95% confidence interval). Make clear which	10-11

For peer review only - http://bmjopen.bmj.com/site/about/guidelines.xhtml
For peer review only

		confounders were adjusted for and why they were included	
		(*b*) Report category boundaries when continuous variables were categorized	10-11
		(*c*) If relevant, consider translating estimates of relative risk into absolute risk for a meaningful time period	NA
Other analyses	17	Report other analyses done—eg analyses of subgroups and interactions, and sensitivity analyses	12
**Discussion**			
Key results	18	Summarise key results with reference to study objectives	13
Limitations	19	Discuss limitations of the study, taking into account sources of potential bias or imprecision. Discuss both direction and magnitude of any potential bias	15-16
Interpretation	20	Give a cautious overall interpretation of results considering objectives, limitations, multiplicity of analyses, results from similar studies, and other relevant evidence	13-15
Generalisability	21	Discuss the generalisability (external validity) of the study results	16
**Other information**			
Funding	22	Give the source of funding and the role of the funders for the present study and, if applicable, for the original study on which the present article is based	5

*Give information separately for exposed and unexposed groups.

Note: An Explanation and Elaboration article discusses each checklist item and gives methodological background and published examples of transparent reporting. The STROBE checklist is best used in conjunction with this article (freely available on the Web sites of PLoS Medicine at http://www.plosmedicine.org/, Annals of Internal Medicine at http://www.annals.org/, and Epidemiology at http://www.epidem.com/). Information on the STROBE Initiative is available at http://www.strobe-statement.org.

For peer review only

BMJ Open

Associations between maternal characteristics and pharmaceutical treatment of gestational diabetes: an analysis of the UK Born in Bradford (BiB) cohort study

Journal:	*BMJ Open*
Manuscript ID	bmjopen-2021-053753.R1
Article Type:	Original research
Date Submitted by the Author:	20-Aug-2021
Complete List of Authors:	Martine-Edith, Gilberte; Loughborough University,<br>Johnson, William; Loughborough University<br>Hunsicker, Eugenie; Loughborough University<br>Hamer, Mark; University College London<br>Petherick, Emily; Loughborough University
**Primary Subject Heading**:	Epidemiology
Secondary Subject Heading:	Diabetes and endocrinology, Public health
Keywords:	Diabetes in pregnancy < DIABETES & ENDOCRINOLOGY, EPIDEMIOLOGY, PUBLIC HEALTH

SCHOLARONE™
Manuscripts

I, the Submitting Author has the right to grant and does grant on behalf of all authors of the Work (as defined in the below author licence), an exclusive licence and/or a non-exclusive licence for contributions from authors who are: i) UK Crown employees; ii) where BMJ has agreed a CC-BY licence shall apply, and/or iii) in accordance with the terms applicable for US Federal Government officers or employees acting as part of their official duties; on a worldwide, perpetual, irrevocable, royalty-free basis to BMJ Publishing Group Ltd ("BMJ") its licensees and where the relevant Journal is co-owned by BMJ to the co-owners of the Journal, to publish the Work in this journal and any other BMJ products and to exploit all rights, as set out in our licence.

The Submitting Author accepts and understands that any supply made under these terms is made by BMJ to the Submitting Author unless you are acting as an employee on behalf of your employer or a postgraduate student of an affiliated institution which is paying any applicable article publishing charge ("APC") for Open Access articles. Where the Submitting Author wishes to make the Work available on an Open Access basis (and intends to pay the relevant APC), the terms of reuse of such Open Access shall be governed by a Creative Commons licence – details of these licences and which Creative Commons licence will apply to this Work are set out in our licence referred to above.

Other than as permitted in any relevant BMJ Author's Self Archiving Policies, I confirm this Work has not been accepted for publication elsewhere, is not being considered for publication elsewhere and does not duplicate material already published. I confirm all authors consent to publication of this Work and authorise the granting of this licence.

For peer review only

Associations between maternal characteristics and pharmaceutical treatment of gestational diabetes: an analysis of the UK Born in Bradford (BiB) cohort study

Gilberte Martine-Edith[1], William Johnson[1], Eugenie Hunsicker[2], Mark Hamer[3], and Emily S Petherick[1]

[1]School of Sport, Exercise and Health Sciences, Loughborough University, Loughborough, UK

[2]School of Science, Loughborough University, Loughborough, UK

[3]Institute of Sport, Exercise and Health, Division Surgery Interventional Science, University College London, London, UK

Corresponding author: William Johnson, School of Sport, Exercise and Health Sciences, Loughborough University, Epinal Way, LE11 3TU, Loughborough, UK. E-mail: w.o.johnson@lboro.ac.uk

For peer review only - http://bmjopen.bmj.com/site/about/guidelines.xhtml

Abstract

Objectives To identify the maternal characteristics associated with pharmaceutical treatment of gestational diabetes mellitus (GDM)

Design Prospective birth cohort study

Setting Bradford, UK

Participants 762 women from the Born in Bradford (BiB) cohort who were treated for GDM in a singleton pregnancy. BiB cohort participants were recruited from 2007 until 2010. All women booked for delivery were screened for GDM between 26 and 28 weeks of gestation using a 75g 2-hour oral glucose tolerance test (OGTT).

Outcome measure GDM treatment type: lifestyle changes advice (lifestyle changes), lifestyle changes advice with supplementary insulin (insulin) and lifestyle changes advice with supplementary metformin (metformin)

Results 32% of women were prescribed lifestyle changes advice alone while 68% were offered supplemental pharmaceutical treatment. The odds of receiving pharmaceutical treatment relative to lifestyle changes advice alone were increased for mothers who were obese (OR 4.6 (95% CI 2.8, 7.5)), those who smoked (2.6 (1.2, 5.5)) and had higher fasting glucose levels at OGTT (2.1 (1.6, 2.7)). The odds of being prescribed pharmaceutical treatment rather than lifestyle changes advice were lower for Pakistani women (OR 0.7 (95% CI 0.4, 1.0)) than White British women. Relative to insulin treatment, metformin was more likely to be offered to obese women than normal weight women (RRR 3.2 (1.3, 7.8) and less likely to be prescribed to women with higher fasting glucose concentrations at OGTT (RRR (0.3 (0.2, 0.6)).

For peer review only

Conclusions In the BiB cohort, GDM pharmaceutical treatment tended to be prescribed to women who were obese, White British, who smoked and had more severe hyperglycaemia. The characteristics of metformin-treated mothers differed from those of insulin-treated mothers as they were more likely to be obese but had lower glucose concentrations at diagnosis.

For peer review only

For peer review only - http://bmjopen.bmj.com/site/about/guidelines.xhtml

Strengths and limitations of this study

- This study was based on a large sample of women diagnosed with GDM in a centre where universal GDM screening is in place
- Data used for this study captured a key transitional period in GDM management as metformin was introduced as an additional pharmaceutical treatment option
- The mainly bi-ethnic nature of the sample allowed for the exploration of ethnic differences in GDM treatment between Pakistani and White British women
- The generalisability of the findings might be limited by the fact that this was a single-centre observational study
- The number of women treated with supplemental metformin was relatively small compared to the two other treatment types

For peer review only

For peer review only - http://bmjopen.bmj.com/site/about/guidelines.xhtml

Introduction

Gestational diabetes mellitus (GDM) is one of the most common complications of pregnancy [1]. In 2019, the International Diabetes Federation estimated that 13.2% of pregnancies, or 17 million live births, were affected by GDM worldwide [2]. The reported prevalence of GDM is 5% in the United Kingdom (UK) [3]. Ethnicity is a risk factor for GDM and in particular, South Asian (SA) women have been shown to have a higher risk for GDM than White women [4–6]. The public health significance of GDM lies in the intergenerational cycle of diabetes and obesity risk it perpetuates as GDM is associated with both maternal complications (e.g. pre-eclampsia, caesarean delivery) and health risks for the offspring (e.g. macrosomia, childhood obesity) [7].

Guidelines for initial GDM management recommend lifestyle changes (dietary and exercise advice) [8,9]. While these changes are largely effective, hyperglycaemia persists for 15-30% of women and supplemental pharmacological treatment is required [8]. Historically, subcutaneous insulin was the first-line pharmacological agent [8]. However, metformin has been increasingly accepted following the Metformin in Gestational diabetes (MiG) trial that validated it as a safe alternative to insulin [10], despite uncertainties regarding its long-term effects on offspring health [11]. In the UK, both the 2008 and 2015 National Institute for Health and Care Excellence (NICE) guidelines initially recommend metformin for GDM treatment and insulin is suggested when metformin is contraindicated, not tolerated or ineffective [9].

With the aim to inform clinical management of GDM, previous research has investigated the characteristics associated with the need for supplemental pharmacological treatment in

For peer review only - http://bmjopen.bmj.com/site/about/guidelines.xhtml

mothers with GDM [12–18]. High maternal body mass index (BMI), history of GDM, advanced age and adverse oral glucose tolerance test (OGTT) were amongst factors increasing the probability of receiving pharmacological treatment compared to lifestyle changes advice alone. However, there is still limited evidence of the associations between maternal characteristics and GDM pharmaceutical treatment in the UK [17–21]. Also, despite the known differences in the risk of GDM between SA and White women, the differences in their risk for GDM pharmaceutical treatment relative to lifestyle changes advice remain largely under researched [13,22].

Using a largely bi-ethnic UK birth cohort that included women with GDM treated both before and after metformin introduction, this study aimed to identify the maternal characteristics associated with GDM pharmacological treatment.

Methods

Study

Born in Bradford (BiB) is a longitudinal prospective birth cohort study [23]. Bradford, a city in the north of England, constitutes a multi-ethnic population of more than 500,000 individuals, with 20% of the population of South Asian origin. Data were collected between 2007 and 2010 from 12,453 women (and their partners and offspring) booked for delivery at the Bradford Royal Infirmary [24]. Ethical approval for the study was granted by Bradford Research Ethics Committee (Ref 07/H1302/112).

For peer review only - http://bmjopen.bmj.com/site/about/guidelines.xhtml

Patient and public involvement

This was a secondary analysis of data from the BiB cohort. BiB has a number of established community advisory groups who are involved in the design, conduct, reporting and dissemination of findings from the Born in Bradford research programme.

Sample

Our sample comprised 762 women with data on maternal characteristics (Figure 1). Cohort participants diagnosed with GDM in a singleton pregnancy were included if they received (i) lifestyle changes advice only, (ii) lifestyle changes advice with supplementary insulin or (iii) lifestyle changes advice supplemented by metformin. We excluded GDM treatment combinations (e.g., lifestyle changes advice supplemented by both metformin and insulin treatment) that did not yield sufficient numbers for meaningful analyses to be conducted. Participants with GDM for whom treatment was not recorded were excluded. If mothers had more than one singleton pregnancy affected and treated for GDM during the study, we only included the first pregnancy. Singleton pregnancies not affected by GDM and higher order pregnancies (twins, triplets) whether or not affected by GDM were excluded from the study, as were women with pre-existing diabetes.

Screening and diagnosis of GDM

All women enrolled in the BiB study were offered GDM screening. This was conducted between 26 and 28 weeks of gestation using the 2-hour 75g OGTT and 80% of women attended their appointment [24]. Diagnosis of GDM was made using the modified 1999 World Health Organisation (WHO) criteria in accordance with local recommendations at the

For peer review only - http://bmjopen.bmj.com/site/about/guidelines.xhtml

time of recruitment (fasting glucose concentration ≥6.1mmol/L and/or 2-hour post-load glucose ≥7.8mmol/L) [25].

Management and treatment of GDM

Local procedure meant that all women were referred to the joint obstetric diabetes clinic following a diagnosis of GDM. Women were educated in dietary and exercise changes and capillary glucose monitoring. Individualized dietary recommendations were provided by a dietician and daily walking for at least 30 minutes was recommended. If glucose targets were achieved after a week (fasting plasma glucose: 4.0-5.5mmol/L; 2-hour postprandial: ≤7.5mmol/L), lifestyle changes were continued without additional pharmacological treatment. If hyperglycaemia persisted, treatment was supplemented with insulin injections until delivery in the first part of the study (04/2007-03/2009). Following metformin introduction (04/2009), both insulin injections and metformin tablets (850 mg, twice daily) were pharmacological prescription options.

Study outcome: GDM treatment type

The three reported treatment options evaluated in our study were: counselling for lifestyle changes, insulin and metformin. Lifestyle changes consisted of diet and exercise. Insulin and metformin groups included women who initially received lifestyle changes advice followed by supplementary insulin and metformin treatment, respectively.

For peer review only

Maternal characteristics

Socio-demographic characteristics

Seven socio-demographic characteristics were considered: age at childbirth, marital and cohabitation status, ethnicity (White British, Pakistani, other), employment status (previously, currently, or never employed), migration status, educational levels, and parity. These were self-reported using interviewer-administered questionnaires at booking conducted in English or South Asian languages (e.g., Bengali, Punjabi). Ethnicity was grouped according to the UK Office of National Statistics guidelines [26]. Education levels corresponded to ≤five General Certificate of Secondary Education (GCSE) qualification, A level equivalent, higher than A level and other/unknown. Migration status was classified in two groups: mother was born in the UK or moved to the UK at ≤five years old and mother moved to the UK >five years of age. Marital and cohabitation status was defined as married and living with a partner, not married and living with a partner or not living with a partner.

Lifestyle and health characteristics

Nine lifestyle and health variables were analysed: BMI at booking, smoking during pregnancy (yes/no), physical activity levels, family history of diabetes (yes/no), history of GDM before the study (yes/no), pre-existing hypertension (yes/no), gestational age and blood glucose concentrations at OGTT (fasting and 2-hour post-load) and start date of treatment relative to metformin introduction (before/after). Maternal BMI was obtained from height and weight measurements conducted at recruitment using Leicester Height Measure and SECA digital scales. Family history of diabetes, history of GDM and pre-existing hypertension were self-reported. Gestational age was recorded, and plasma glucose levels were measured at OGTT using a glucose oxidase method. Maternal physical activity levels (inactive,

For peer review only - http://bmjopen.bmj.com/site/about/guidelines.xhtml

moderately inactive, moderately active, active) were self-reported using the UK General Practice Physical Activity Questionnaire [27].

Statistical analysis

Analyses were based on two time periods to account for the fact that metformin was used for GDM treatment in the study from April 2009 onwards, which is two years after the first women with GDM were offered lifestyle changes advice with or without insulin treatment in the cohort.

Overall study period: April 2007 – February 2011

Descriptive analysis

Using the whole study sample, we considered two treatment types: lifestyle changes advice and pharmaceutical treatment (i.e., insulin- and metformin-treated women were grouped). Differences in maternal characteristics between women receiving lifestyle changes advice alone and those receiving supplemental pharmaceutical treatment were explored using the Mann-Whitney U test for continuous variables and Chi-square (or Fisher's exact) test for categorical variables. The Holm-Bonferroni correction adjusted for multiple testing [28,29].

Regression analysis

Variable selection for the binary logistic regression model was conducted using the least absolute shrinkage and selection operator (LASSO) which shrinks less stable coefficients exactly to zero, allowing for the selection of a more parsimonious model [30]. For each

For peer review only - http://bmjopen.bmj.com/site/about/guidelines.xhtml

maternal characteristic selected through LASSO, a regression model was fitted to assess the unadjusted relationships between maternal characteristic and GDM pharmaceutical treatment, relative to lifestyle changes advice. The associations between maternal characteristics and GDM treatment were further assessed in a fully adjusted model, including all maternal characteristics.

Sensitivity analysis

Given the higher risk for insulin resistance and GDM in Pakistani women compared to White British women in the BiB cohort [31], we reproduced the whole sample analysis but stratified by ethnicity, to evaluate whether the associations between maternal characteristics and GDM pharmaceutical treatment were influenced by ethnicity. Differences in maternal characteristics between White British and Pakistani women were also examined.

Period after metformin introduction: April 2009 – February 2011

Descriptive analysis

Using the subsample of women who started GDM treatment after metformin introduction, we considered three treatment types: lifestyle changes advice, insulin, and metformin. The differences in the LASSO-selected maternal characteristics were examined by GDM treatment type. The Kruskal-Wallis test was used for continuous variables and the Chi-square (or Fisher's exact) test was used for categorical variables. The Holm-Bonferroni correction adjusted for multiple testing [28,29].

For peer review only - http://bmjopen.bmj.com/site/about/guidelines.xhtml

Regression analysis

The relationships between maternal characteristics and insulin and metformin treatment were evaluated compared to lifestyle changes advice alone, in a multinomial logistic regression model including the LASSO-selected characteristics. The same multinomial logistic regression was fitted but using insulin as the reference group, to examine the maternal characteristics associated with metformin rather than insulin (the associations between maternal characteristics and lifestyle changes advice relative to insulin were omitted).

Analyses were conducted using R (R 3.4.1 & R Studio 1.0.153 for Windows) and Stata/SE software (Stata/SE 15 for Windows; StataCorp, College Station, TX, USA).

Results

A total of 844 women were diagnosed with GDM in a singleton pregnancy. 82 women who did not meet treatment inclusion criteria were excluded, leading to a sample of 762 women (Figure 1).

<u>Overall study period: lifestyle changes vs pharmaceutical treatment</u>

32% of women received lifestyle changes advice alone and 68% received supplemental pharmacological treatment during the study (Table 1). Women who were prescribed pharmacological treatment were older at childbirth (median age: 31.7 years (interquartile range, IQR: 7.6) compared to women receiving lifestyle changes advice (29.9 years (8.1)), they were more hyperglycaemic at OGTT and had higher obesity rates (41.7% vs 19.0%).

For peer review only - http://bmjopen.bmj.com/site/about/guidelines.xhtml

These differences remained statistically significant after accounting for multiple testing (Table 1).

A total of 12 maternal characteristics were selected via LASSO and these were included in the regression analysis (Table 2). Unadjusted analysis showed that obese women had five times the odds of being reported to have been offered pharmaceutical treatment (OR 4.6 (95% CI 2.8, 7.5)) than lifestyle changes advice. The odds of pharmaceutical treatment compared to lifestyle changes advice were 2.6 (1.2, 5.5) times higher for women who smoked during pregnancy and 2.1 (1.6, 2.7) greater for women who had higher fasting glucose concentrations at OGTT. Relative to White British women, Pakistani women were predicted to have lower odds of being prescribed pharmaceutical treatment (OR 0.7 (0.4, 1.0)) (Table 2). Obesity and smoking were less prevalent among Pakistani women than White British women (Supplementary Table 1).

Fully adjusted analyses confirmed that obesity, smoking and higher glucose concentrations at diagnosis were associated with higher odds of pharmaceutical treatment although the estimates were attenuated (Table 2). Adjusting for fasting glucose weakened the relationships between obesity and pharmaceutical treatment. Adjustments for ethnicity brought the estimates for smoking closer towards the null.

The sensitivity analysis showed that for both White British and Pakistani women, higher glucose concentrations at OGTT and obesity were associated with an increase in the odds of being prescribed pharmaceutical treatment relative to lifestyle changes advice alone (Supplementary Table 2).

For peer review only - http://bmjopen.bmj.com/site/about/guidelines.xhtml

Period after metformin introduction: lifestyle changes advice vs insulin vs metformin

After metformin introduction, 31.1% of women received lifestyle changes advice alone, 50.5% were prescribed supplemental insulin and 18.4% were offered supplemental metformin (Table 3). Mothers in the lifestyle changes group were more likely to be younger, less hyperglycaemic and have a lower BMI than women receiving supplemental insulin or metformin.

Relative to lifestyle changes advice, the risk of insulin treatment was 2.3 times higher for both obese women and women with higher fasting glucose concentrations at OGTT (Table 4). The risk of insulin treatment relative to lifestyle changes advice was also higher for women who smoked during pregnancy compared to those who did not smoke. Supplemental metformin treatment rather than lifestyle changes advice alone was 7.3 times (2.7, 20.0) more likely for obese women.

Compared to insulin treatment, the risk of metformin was three times higher for both obese compared to normal weight women and Pakistani women compared to White British women (Table 4). Higher fasting glucose concentrations at OGTT were associated with a lower risk (RRR 0.3 (0.2, 0.6)) of a record of receiving metformin treatment relative to insulin.

Discussion

Our study showed that obesity, smoking and higher glucose concentrations at OGTT were key maternal characteristics associated with supplemental pharmaceutical treatment

For peer review only - http://bmjopen.bmj.com/site/about/guidelines.xhtml

compared to lifestyle changes advice alone. Ethnic differences were also identified as, relative to White British women, Pakistani women were less likely to receive pharmaceutical treatment as a whole than lifestyle changes advice. Among women who received pharmaceutical treatment, metformin was more likely to be prescribed to obese women than normal weight women and to Pakistani women than White British women. Women who were more hyperglycaemic at diagnosis were more likely to be prescribed insulin rather than metformin.

Lifestyle changes advice supplemented by pharmaceutical treatment was the most common form of GDM management in our study. This contrasted with previous studies in which mothers with GDM were more frequently managed with lifestyle changes advice [12,14–17,32–34]. These disparities could be due to differences in GDM diagnostic criteria: the modified 1999 WHO criteria in our study used higher fasting glucose thresholds at OGTT but lower 2-hour thresholds than other criteria in by previous studies [12,16,34]. Additionally, the higher rates of pharmaceutical treatment in our study could reflect the higher risk profile of the BiB population and also, the high levels of deprivation in Bradford [24] could have limited health literacy and the adherence to lifestyle changes advice [35].

Obesity, smoking during pregnancy and glucose concentrations at OGTT were the maternal characteristics most strongly associated with GDM supplemental pharmaceutical treatment in comparison to lifestyle changes advice alone. Previous research has also reported BMI as a risk factor for GDM pharmaceutical treatment, notably insulin. Although the specificity of these studies largely varied (e.g., location, sample size, screening methods, diagnostic thresholds), they consistently showed that as maternal BMI increased, so did the risk of being

For peer review only - http://bmjopen.bmj.com/site/about/guidelines.xhtml

treated with insulin [12–15,32,34,36–38]. Regarding the associations between smoking and GDM treatment, some studies showed that more smokers were treated with insulin than lifestyle changes [15,33,39], whilst others found an opposite relationship [38,40], although the differences between groups in these studies were not reported to be statistically significant. A more recent study has reported that smoking was associated with a higher risk of insulin treatment, although this was relative to women without GDM and women with GDM not requiring insulin treatment combined in the same control group [41].

We hypothesise that the mechanisms explaining the associations between obesity, smoking, glucose concentrations at OGTT and GDM pharmaceutical treatment in our sample are closely related to obesity- and smoking-induced insulin resistance. Obesity may alter the functioning of pancreatic β-cells and exacerbates insulin resistance (which is already increased as a result of pregnancy) [22,38,42–45]. Smoking has also been associated with insulin resistance, via processes including hormonal secretions (e.g. growth hormone) that counteract insulin action [46,47]. Thus, although there was no direct measure of insulin resistance in this study, it is possible that women who were obese or smoked during pregnancy had a higher degree of insulin resistance. Additionally, in line with other studies [34,48–50], we found that women who were prescribed pharmaceutical treatment were more likely to be more severely hyperglycaemic compared to women who received lifestyle changes advice alone. As increases in insulin resistance and β-cell dysfunction can further lead to higher glucose concentrations at the OGTT [51–53], the severity of insulin resistance and its associated greater severity of hyperglycaemia in obese women and those who smoked could have been such that lifestyle changes advice alone were insufficient to achieve glucose targets. In that sense, our results accurately reflect clinical practice in Bradford as the decision to prescribe pharmaceutical treatment was based on the finding of glucose levels

For peer review only - http://bmjopen.bmj.com/site/about/guidelines.xhtml

higher than the glucose targets. Further, what our study suggests is that the severity of hyperglycaemia may mediate the relationships between maternal obesity and smoking and GDM pharmaceutical treatment. This was confirmed by individual adjustment for fasting glucose which attenuated the relationships between obesity and GDM pharmaceutical treatment. although this attenuation was less evident for the relationships between smoking and GDM pharmaceutical treatment possibly due to the low proportion of smokers.

Another important finding of this study is that, relative to White British women, Pakistani women were predicted to have a lower risk for pharmaceutical treatment (when insulin and metformin treatment were grouped) compared to lifestyle changes alone. This may seem counterintuitive given SA women are more prone to insulin resistance than White European women due to a greater susceptibility to store adipose tissue viscerally rather than subcutaneously [6,54]. Wong and Jalaludin (2012) and Wong (2011) have also described that SA women had a lower risk to be prescribed with supplemental insulin rather than lifestyle changes advice alone than Anglo-Europeans. The authors suggested that this may be due to differences between the two ethnic groups in the placental factors impacting, late in the pregnancy, on the severity of insulin resistance [13,22]. Underlying ethnic differences in dietary habits during pregnancy and adherence to treatment could also be contributing factors [22]. In the context of our study however, Pakistani mothers were less likely to report smoking and had lower BMI compared to White British women which was consistent with previous research in the BiB cohort [24,55,56]. This, combined with the fact that obesity and smoking were strongly associated with GDM pharmaceutical treatment in our study, may explain why Pakistani women were less likely to receive pharmaceutical treatment rather than lifestyle changes advice alone compared to White British women. The stratified analysis however showed that higher maternal BMI and glucose concentration at OGTT were

For peer review only - http://bmjopen.bmj.com/site/about/guidelines.xhtml

For peer review only

associated with a higher risk for pharmaceutical treatment relative to lifestyle changes advice, irrespective of maternal ethnicity.

The addition of metformin to the set of pharmacological options was not associated, at the time of the study, with any substantial shift in GDM management as insulin remained the most common prescribed treatment. Nevertheless, we found that, obese women were more likely to be treated with metformin rather than insulin which is in line with a study by McGrath *et al.* (2018). This perhaps is the result of clinical decision-making as metformin, compared to insulin, has been associated with lower weight gain [57] thus metformin could preferably be given to women with higher BMI. Further, we found that women with more severe hyperglycaemia were more likely to be prescribed insulin rather than metformin, which corroborated previous research [58–60]. As metformin is believed to act less rapidly than insulin [18], it may be that in our study, even after metformin introduction, women with a higher severity of hyperglycaemia were preferentially prescribed insulin to promptly restore euglycaemia. Thus, it is somewhat surprising that Pakistani mothers, characteristically more hyperglycaemic and with lower BMI than White British women, were predicted to have a higher risk for metformin treatment compared to insulin than White British women. This may reflect individual treatment preference for metformin treatment as insulin injections are considered by mothers with GDM to be invasive and burdensome [61] and can be associated with social stigma within SA communities [62]. More research regarding the ethnic differences between metformin- and insulin-treated mothers with GDM would be needed to ascertain this finding.

For peer review only - http://bmjopen.bmj.com/site/about/guidelines.xhtml

The main strength of this study is that the findings are based on a large sample of women diagnosed with GDM from a cohort where universal GDM screening was in place. The data originated from a single diabetes clinic in the UK managed by the same senior clinician and where the same diagnostic criteria and glucose targets for GDM management were used throughout the study. This minimised bias related to differences in clinical practice and decisions between clinics. Another strength of our study is that, unlike previous studies that explored maternal characteristics of GDM treatment either before or after metformin introduction, our data captured GDM management both pre- and post-metformin introduction. This allowed for an analysis of the maternal characteristics associated with GDM pharmacological treatment during a key transitional period of changes in GDM management within the BiB cohort. Lastly, the mainly bi-ethnic nature of the BiB cohort enabled the assessment of the effect of being Pakistani, relative to White British, on the risk for GDM pharmaceutical treatment which is particularly important given Pakistani mothers have a higher risk of developing GDM itself.

Our findings are however limited by the relatively small sample of women treated with metformin at the time of the BiB study compared to the other treatment types which means that our results must be interpretated with caution. We acknowledge that the generalisability of our results may be limited by the fact that this is a single-centre observational study, although our findings remained largely consistent with previous research.

To conclude, in the UK BiB cohort, women who received GDM supplemental pharmaceutical treatment rather than lifestyle changes advice alone were more likely to be obese, smokers, more hyperglycaemic and White British. Among women who received

For peer review only

For peer review only - http://bmjopen.bmj.com/site/about/guidelines.xhtml

pharmaceutical treatment, the risk for metformin treatment was higher for Pakistani women and obese women, whilst women who were more hyperglycaemic were more likely to be prescribed insulin. Evaluation of the relationships between GDM treatment and maternal or offspring outcomes in the BiB cohort would thus have to account for the maternal determinants of GDM pharmaceutical treatment identified in this study.

For peer review only

Acknowledgements

Born in Bradford is only possible because of the enthusiasm and commitment of the children and parents in BiB. We are grateful to all the participants, health professionals, schools and researchers who have made Born in Bradford happen.

Declarations

Funding

This research was funded by Loughborough University and supported by the National Institute for Health Research (NIHR) Leicester Biomedical Research Centre. E.S.P. and W.J. acknowledge support from the National Institute for Health Research (NIHR) Leicester Biomedical Research Centre, which is a partnership between University Hospitals of Leicester NHS Trust, Loughborough University, and the University of Leicester. W.J. is supported by a UK Medical Research Council (MRC) New Investigator Research Grant (MR/P023347/1). Born in Bradford received funding from a Wellcome Trust infrastructure grant (WT101597MA), the National Institute for Health Research under its Collaboration for Applied Health Research and Care (CLAHRC) (IS-CLA-0113-10020). The NIHR Clinical Research Network which provided research delivery support for this study.

The views expressed in this paper are those of the authors and not necessarily those of the NIHR.

Authors' contribution

G.M-E. wrote the manuscript and was responsible for the acquisition, analysis, and interpretation of data. E.S.P. and W.J. revised the manuscript and contributed to the

For peer review only - http://bmjopen.bmj.com/site/about/guidelines.xhtml

acquisition, analysis, and interpretation of data. E.H. contributed to the analysis and interpretation of data and reviewed the manuscript. M.H. reviewed the manuscript. G.M-E., W.J. and E.S.P. are guarantors of this work and, as such, had full access to all the data in the study and take responsibility for the integrity of the data and the accuracy of the data analysis.

Declarations of interest

None

Data availability

Scientists are encouraged and able to use BiB data. Data requests are made to the BiB executive using the form available from the study website http://www.borninbradford.nhs.uk. Guidance for researchers and collaborators, the study protocol and the data collection schedule are all available via the website. All requests are carefully considered and accepted where possible.

Ethics approval

Ethical approval for the study was granted by Bradford Research Ethics Committee (Ref 07/H1302/112).

Informed consent

All participants provided written consent for the BiB study.

For peer review only

References

1 American Diabetes Association. Diagnosis and Classification of Diabetes Mellitus. *Diabetes Care* 2008;**31**:S55–60. doi:10.2337/dc08-S055

2 International Diabetes Federation. *IDF Diabetes Atlas 2019*. 2019. http://www.idf.org/about-diabetes/facts-figures

3 Diabetes UK. Us, diabetes and a lot of facts and stats. London: 2019. www.diabetes.org.uk

4 Farrar D, Fairley L, Santorelli G, *et al.* Association between hyperglycaemia and adverse perinatal outcomes in south Asian and white British women: Analysis of data from the Born in Bradford cohort. *Lancet Diabetes Endocrinol* 2015;**3**:795–804. doi:10.1016/S2213-8587(15)00255-7

5 Anand SS, Gupta M, Teo KK, *et al.* Causes and consequences of gestational diabetes in South Asians living in Canada: results from a prospective cohort study. *C Open* 2017;**5**:E604–11. doi:10.9778/cmajo.20170027

6 Makgoba M, Savvidou MD, Steer PJ. An analysis of the interrelationship between maternal age, body mass index and racial origin in the development of gestational diabetes mellitus. *BJOG An Int J Obstet Gynaecol* 2012;**119**:276–82. doi:10.1111/j.1471-0528.2011.03156.x

7 Chen L, Mayo R, Chatry A, *et al.* Gestational Diabetes Mellitus: Its Epidemiology and Implication beyond Pregnancy. *Curr Epidemiol Reports* 2016;**3**:1–11. doi:10.1007/s40471-016-0063-y

8 American Diabetes Association. Management of diabetes in pregnancy: Standards of medical care in diabetes. *Diabetes Care* 2019;**42**:S165–72. doi:10.2337/dc19-S014

9 National Institute for Health and Care Excellence. Diabetes in pregnancy : management from preconception to the postnatal period. 2015. https://www.nice.org.uk/guidance/ng3

10 Rowan JA, Hague WM, Gao W, *et al.* Metformin versus Insulin for the Treatment of Gestational Diabetes. *N Engl J Med* 2008;**358**:2003–15. doi:10.1056/NEJMoa0707193

11 Lindsay RS, Loeken MR. Metformin use in pregnancy: promises and uncertainties. *Diabetologia* 2017;**60**:1612–9. doi:10.1007/s00125-017-4351-y

12 Barnes RA, Wong T, Ross GP, *et al.* A novel validated model for the prediction of insulin therapy initiation and adverse perinatal outcomes in women with gestational diabetes mellitus. *Diabetologia* 2016;**59**:2331–8. doi:10.1007/s00125-016-4047-8

13 Wong VW, Jalaludin B. Gestational diabetes mellitus: Who requires insulin therapy? *Aust New Zeal J Obstet Gynaecol* 2011;**51**:432–6. doi:10.1111/j.1479-828X.2011.01329.x

14 Aktun LH, Yorgunlar B, Karaca N, *et al.* Predictive Risk Factors in the Treatment of Gestational Diabetes Mellitus. *Clin Med Insights Women's Heal* 2015;**8**:CMWH.S31564. doi:10.4137/cmwh.s31564

15 González-Quintero VH, Istwan NB, Rhea DJ, *et al.* Antenatal Factors Predicting

For peer review only

Subsequent Need for Insulin Treatment in Women with Gestational Diabetes. *J Women's Heal* 2008;**17**:1183–7. doi:10.1089/jwh.2007.0667

16 Zhang Y, Shao J, Li F, *et al.* Factors in Gestational Diabetes Mellitus Predicting the Needs for Insulin Therapy. *Int J Endocrinol* 2016;**2016**:1–5. doi:10.1155/2016/4858976

17 Ali A, Shastry S, Nithiyananthan R, *et al.* Gestational diabetes–Predictors of response to treatment and obstetric outcome. *Eur J Obstet Gynecol Reprod Biol* 2018;**220**:57–60. doi:10.1016/j.ejogrb.2017.11.014

18 Khin MO, Gates S, Saravanan P. Predictors of metformin failure in gestational diabetes mellitus (GDM). *Diabetes Metab Syndr Clin Res Rev* 2018;**12**:405–10. doi:10.1016/j.dsx.2018.01.003

19 Gandhi P, Bustani R, Madhuvrata P, *et al.* Introduction of metformin for gestational diabetes mellitus in clinical practice: Has it had an impact? *Eur J Obstet Gynecol Reprod Biol* 2012;**160**:147–50. doi:10.1016/j.ejogrb.2011.11.018

20 Balani J, Hyer S, Johnson A, *et al.* Pregnancy outcomes after metformin treatment for gestational diabetes: a case-control study. *Obstet Med* Published Online First: 2012. doi:10.1258/om.2012.110092

21 Balani J, Hyer SL, Rodin DA, *et al.* Pregnancy outcomes in women with gestational diabetes treated with metformin or insulin: A case-control study. *Diabet Med* 2009;**26**:798–802. doi:10.1111/j.1464-5491.2009.02780.x

22 Wong VW. Gestational diabetes mellitus in five ethnic groups: A comparison of their clinical characteristics. *Diabet Med* 2012;**29**:366–71. doi:10.1111/j.1464-5491.2011.03439.x

23 Raynor P, Duley L, Small N, *et al.* Born in Bradford, a cohort study of babies born in Bradford, and their parents: Protocol for the recruitment phase. *BMC Public Health* 2008;**8**:1–13. doi:10.1186/1471-2458-8-327

24 Wright J, Small N, Raynor P, *et al.* Cohort profile: The born in bradford multi-ethnic family cohort study. *Int J Epidemiol* 2013;**42**:978–91. doi:10.1093/ije/dys112

25 World Health Organisation. Definition, diagnosis and classification of diabetes mellitus and its complications. Report of a WHO consultation. Part 1: diagnosis and classification of diabetes mellitus. https://apps.who.int/iris/handle/10665/66040

26 Office for National Statistics. Ethnic group statistics: a guide for the collection and classification of ethnicity data - Office for National Statistics. Off. Natl. Stat. 2003.https://www.ons.gov.uk/methodology/classificationsandstandards/measuringequality/ethnicgroupnationalidentityandreligion

27 National Health Service . The General Practice Physical Activity Questionnaire (GPPAQ): A screening tool to assess adult physical activity levels, within primary care. 2009. doi:10.1007/s00330-011-2164-9

28 Holm S. A Simple Sequentially Rejective Multiple Test Procedure. *Scand J Stat* 1979;**6**:65–70.

29 Gaetano J. Holm-Bonferroni sequential correction: An EXCEL calculator (1.1) [Microsoft Excel workbook].

For peer review only - http://bmjopen.bmj.com/site/about/guidelines.xhtml

https://www.researchgate.net/publication/236969037_Holm-Bonferroni_Sequential_Correction_An_EXCEL_Calculator. 2013.

30 Vincent M, Hansen NR. Sparse group lasso and high dimensional multinomial classification. *Comput Stat Data Anal* 2014;**71**:771–86. doi:10.1016/j.csda.2013.06.004

31 West J, Lawlor DA, Fairley L, *et al.* Differences in socioeconomic position, lifestyle and health-related pregnancy characteristics between Pakistani and White British women in the Born in Bradford prospective cohort study: The influence of the woman's, her partner's and their parents' place . *BMJ Open* 2014;**4**. doi:10.1136/bmjopen-2014-004805

32 Koning SH, Scheuneman KA, Lutgers HL, *et al.* Risk stratification for healthcare planning in women with gestational diabetes mellitus. *Neth J Med* 2016;**74**:262–9.

33 Sapienza AD, Francisco RPV, Trindade TC, *et al.* Factors predicting the need for insulin therapy in patients with gestational diabetes mellitus. *Diabetes Res Clin Pract* 2010;**88**:81–6. doi:10.1016/j.diabres.2009.12.023

34 Nishikawa T, Ono K, Hashimoto S, *et al.* One-hour oral glucose tolerance test plasma glucose at gestational diabetes diagnosis is a common predictor of the need for insulin therapy in pregnancy and postpartum impaired glucose tolerance. *J Diabetes Investig* 2018;**9**:1370–7. doi:10.1111/jdi.12848

35 Draffin CR, Alderdice FA, McCance DR, *et al.* Exploring the needs, concerns and knowledge of women diagnosed with gestational diabetes: A qualitative study. *Midwifery* 2016;**40**:141–7. doi:10.1016/j.midw.2016.06.019

36 Pertot T, Molyneaux L, Tan K, *et al.* Can common clinical parameters be used to identify patients who will need insulin treatment in gestational diabetes mellitus? *Diabetes Care* 2011;**34**:2214–6. doi:10.2337/dc11-0499

37 Bakiner O, Bozkirli E, Ozsahin K, *et al.* Risk Factors That can Predict Antenatal Insulin Need in Gestational Diabetes. *J Clin Med Res* 2013;**5**:381–8. doi:10.4021/jocmr1515w

38 Yanagisawa K, Muraoka M, Takagi K, *et al.* Assessment of predictors of insulin therapy in patients with gestational diabetes diagnosed according to the IADPSG criteria. *Diabetol Int* 2016;**7**:440–6. doi:10.1007/s13340-016-0272-0

39 Lemieux L, Ryan EA. The Need for Insulin in Subjects With Impaired Glucose Tolerance of Pregnancy. *Can J Diabetes* 2004;**28**:196–200.https://www.researchgate.net/publication/289150216_The_need_for_insulin_in_subjects_with_impaired_glucose_tolerance_of_pregnancy

40 Akinci B, Celtik A, Yener S, *et al.* Is fasting glucose level during oral glucose tolerance test an indicator of the insulin need in gestational diabetes? *Diabetes Res Clin Pract* 2008;**82**:219–25. doi:10.1016/j.diabres.2008.07.023

41 Kim MK, Han K, You SY, *et al.* Prepregnancy smoking and the risk of gestational diabetes requiring insulin therapy. *Sci Rep* Published Online First: 2020. doi:10.1038/s41598-020-70873-7

42 Pertot T, Molyneaux L, Tan K, *et al.* Can common clinical parameters be used to identify patients who will need insulin treatment in gestational diabetes mellitus?

For peer review only - http://bmjopen.bmj.com/site/about/guidelines.xhtml

Diabetes Care 2011;**34**:2214–6. doi:10.2337/dc11-0499

43 Ketumarn N, Boriboonhirunsarn D. Characteristics of abnormal oral glucose tolerance test in GDM diagnosis and clinical correlation. *J Matern Neonatal Med* 2018;**31**:2109–14. doi:10.1080/14767058.2017.1336224

44 Sapienza AD, Francisco RPV, Trindade TC, *et al.* Factors predicting the need for insulin therapy in patients with gestational diabetes mellitus. *Diabetes Res Clin Pract* 2010;**88**:81–6. doi:10.1016/j.diabres.2009.12.023

45 Catalano PM. Obesity, insulin resistance, and pregnancy outcome. *REPRODUCTION* 2010;**140**:365–71. doi:10.1530/REP-10-0088

46 Artese A, Stamford BA, Moffatt RJ. Cigarette Smoking: An Accessory to the Development of Insulin Resistance. *Am J Lifestyle Med* Published Online First: 2019. doi:10.1177/1559827617726516

47 Sliwinska-Mosson M, Milnerowicz H. The impact of smoking on the development of diabetes and its complications. *Diabetes Vasc Dis Res* 2017;**14**:265–76. doi:10.1177/1479164117701876

48 Ito Y, Shibuya M, Hosokawa S, *et al.* Indicators of the need for insulin treatment and the effect of treatment for gestational diabetes on pregnancy outcomes in Japan. *Endocr J* 2016;**63**:231–7. doi:10.1507/endocrj.EJ15-0427

49 Watanabe M, Katayama A, Kagawa H, *et al.* Risk Factors for the Requirement of Antenatal Insulin Treatment in Gestational Diabetes Mellitus. *J Diabetes Res* 2016;**2016**:1–6. doi:10.1155/2016/9648798

50 Zhang Y, Shao J, Li F, *et al.* Factors in Gestational Diabetes Mellitus Predicting the Needs for Insulin Therapy. *Int J Endocrinol* 2016;**2016**:1–5. doi:10.1155/2016/4858976

51 Abdul-Ghani MA, Matsuda M, Jani R, *et al.* The relationship between fasting hyperglycemia and insulin secretion in subjects with normal or impaired glucose tolerance. *Am J Physiol - Endocrinol Metab* 2008;**295**. doi:10.1152/ajpendo.00674.2007

52 Sokup A, Ruszkowska-Ciastek B, Góralczyk K, *et al.* Insulin resistance as estimated by the homeostatic method at diagnosis of gestational diabetes: Estimation of disease severity and therapeutic needs in a population-based study. *BMC Endocr Disord* 2013;**13**. doi:10.1186/1472-6823-13-21

53 Saisho Y, Miyakoshi K, Tanaka M, *et al.* Beta cell dysfunction and its clinical significance in gestational diabetes. *Endocr J* 2010;**57**:973–80. doi:http://dx.doi.org/10.1507/endocrj.K10E-231

54 Sniderman AD, Bhopal R, Prabhakaran D, *et al.* Why might South Asians be so susceptible to central obesity and its atherogenic consequences? The adipose tissue overflow hypothesis. *Int J Epidemiol* 2007;**36**:220–5. doi:10.1093/ije/dyl245

55 West J, Santorelli G, Whincup PH, *et al.* Association of maternal exposures with adiposity at age 4/5 years in white British and Pakistani children: findings from the Born in Bradford study. *Diabetologia* 2018;**61**:242–52. doi:10.1007/s00125-017-4457-2

For peer review only - http://bmjopen.bmj.com/site/about/guidelines.xhtml

56 Lawlor DA, West J, Fairley L, *et al.* Pregnancy glycaemia and cord-blood levels of insulin and leptin in Pakistani and white British mother-offspring pairs: Findings from a prospective pregnancy cohort. *Diabetologia* 2014;**57**:2492–500. doi:10.1007/s00125-014-3386-6

57 Balsells M, Garcia-Patterson A, Sola I, *et al.* Glibenclamide, metformin, and insulin for the treatment of gestational diabetes: a systematic review and meta-analysis. *Bmj* 2015;**350**:h102–h102. doi:10.1136/bmj.h102

58 McGrath R, Glastras S, Scott E, *et al.* Outcomes for Women with Gestational Diabetes Treated with Metformin: A Retrospective, Case-Control Study. *J Clin Med* 2018;**7**:50. doi:10.3390/jcm7030050

59 Goh JEL, Sadler L, Rowan J. Metformin for gestational diabetes in routine clinical practice. *Diabet Med* 2011;**28**:1082–7. doi:10.1111/j.1464-5491.2011.03361.x

60 Ijas H, Vaarasmaki M, Morin-Papunen L, *et al.* Metformin should be considered in the treatment of gestational diabetes: A prospective randomised study. *BJOG An Int J Obstet Gynaecol* 2010;**118**:880–5. doi:10.1111/j.1471-0528.2010.02763.x

61 Latif L, Hyer S, Shehata H. Metformin effects on treatment satisfaction and quality of life in gestational diabetes. *Br J Diabetes Vasc Dis* 2013;**13**:178–82. doi:10.1177/1474651413493933

62 Kumar K, Greenfield S, Raza K, *et al.* Understanding adherence-related beliefs about medicine amongst patients of South Asian origin with diabetes and cardiovascular disease patients: A qualitative synthesis. *BMC Endocr Disord* Published Online First: 2016. doi:10.1186/s12902-016-0103-0

For peer review only

Table 1 Maternal characteristics by GDM treatment type across the whole study period (2007-2011)

	Lifestyle changes advice (n=244)	Pharmaceutical treatment (n=518)	*p*	*p**	n (%) Missing
**Start date of treatment‡**, n (%)			0.486	>0.999	4 (0.5)
Before metformin introduction (2007-09)	121 (49.6)	241 (47)			
After metformin introduction (2009-11)	123 (50.4)	273 (53)			
**Age at childbirth† (years)**, median (IQR)	29.9 (8.1)	31.7 (7.6)	<0.001	0.001	0
**BMI at booking† (kg/m^2)**, median (IQR)	25.2 (6.0)	28.4 (7.9)	<0.001	<0.001	46 (6.0)
**BMI category at booking‡**, n (%)			<0.001	<0.001	46 (6.0)
Underweight (*BMI<18.5 kg/m^2*)	7 (3.0)	7 (1.4)			
Normal weight (*18.5≤BMI≤24.9 kg/m^2*)	107 (46.1)	122 (25.2)			
Overweight (*25.0≤BMI≤29.9 kg/m^2*)	74 (31.9)	153 (31.6)			
Obese (*BMI ≥30.0 kg/m^2*)	44 (19.0)	202 (41.7)			
**Smoking during pregnancy‡**, n (%)			0.008	0.096	1 (0.1)
Yes	11 (4.5)	53 (10.2)			
No	233 (95.5)	464 (89.8)			
**Parity‡**, n (%)			0.671	>0.999	24 (3.1)
0	88 (37.0)	164 (32.8)			
1	53 (22.3)	119 (23.8)			
2	41 (17.2)	99 (19.8)			
3+	56 (23.5)	118 (23.6)			
**Physical activity levels‡**, n (%)			0.684	>0.999	109 (14)
Inactive	134 (62.3)	285 (65.1)			
Moderately inactive	37 (17.2)	77 (17.6)			
Moderately active	35 (16.3)	56 (12.8)			
Active	9 (4.2)	20 (4.6)			
**Ethnic group‡**, n (%)			0.060	0.540	0
White British	47 (19.3)	140 (27.0)			
Pakistani	152 (62.3)	298 (57.5)			
Other	45 (18.4)	80 (15.4)			
**Migration status‡**, n (%)			0.253	>0.999	13 (1.7)
Born in the UK or moved ≤ 5 years	121 (51.3)	286 (55.7)			
Moved to the UK > 5 years	115 (48.7)	227 (44.2)			
**Marital and cohabitation status‡**, n (%)			0.239	>0.999	0
Married and living with partner	198 (81.1)	411 (79.3)			
Not married and living with partner	21 (8.6)	64 (12.4)			
Not living with partner	25 (10.2)	43 (8.3)			
**Highest educational qualification‡**, n (%)			0.528	>0.999	4 (0.5)
5 GCSE equivalent or less	124 (51.0)	273 (53.0)			
A-level equivalent	28 (11.5)	57 (11.1)			
Higher than A-level	76 (31.3)	141 (27.4)			
Other/Unknown	15 (6.2)	44 (8.5)			
**Family history of diabetes‡**, n (%)			0.010	0.100	63 (8.3)
Yes	128 (57.7)	323 (67.7)			
No	94 (42.3)	154 (32.3)			

For peer review only - http://bmjopen.bmj.com/site/about/guidelines.xhtml

**Pre-existing hypertension§** , n (%)			0.563	>0.999	53 (7.0)
Yes	3 (1.3)	10 (2.1)			
No	228 (98.7)	468 (97.9)			
**History of GDM before the study‡** , n (%)			0.075	0.600	93 (12)
Yes	10 (4.6)	38 (8.4)			
No	207 (95.4)	414 (91.6)			
**Mother's employment status‡** , n (%)			0.008	0.096	0
Currently employed	90 (36.9)	203 (39.2)			
Previously employed	55 (22.5)	159 (30.7)			
Never employed	99 (40.6)	156 (30.1)			
**Gestational age at OGTT† (weeks),** median (IQR)	26.4 (1.6)	26.3 (0.8)	0.006	0.078	13 (1.7)
**Fasting glucose concentrations at OGTT† (mmol/L)**, median (IQR)	4.7 (0.7)	5.1 (1.1)	<0.001	<0.001	13 (1.7)
**2h post-load glucose concentrations at OGTT† (mmol/L)**, median (IQR)	8.2 (0.8)	8.6 (1.6)	<0.001	<0.001	13 (1.7)

A-level: UK highest qualification in high school; BMI: body mass index; GCSE: general certificate of secondary education;
OGTT: oral glucose tolerance test
Continuous data presented as median and interquartile range (IQR).
Categorical data presented as frequencies and percentages.
*Adjusted *P*-value after Holm-Bonferroni correction
†Mann-Whitney U test
‡Chi-square test

For peer review only - http://bmjopen.bmj.com/site/about/guidelines.xhtml

Table 2 Associations between maternal characteristics and pharmaceutical treatment of GDM relative to lifestyle changes advice

	**Pharmaceutical treatment (n=372)**			
	Unadjusted OR (95% CI)	*p*	*Adjusted OR (95% CI)*	*p*
**Mother age at childbirth (years)**	1.1 (1.0, 1.1)	<0.001	1.1 (1.0, 1.1)	<0.001
**BMI categories at booking (kg/m^2)**				
Normal weight	***Reference***		***Reference***	
Underweight	0.8 (0.2, 2.4)	0.663	1.2 (0.4, 4.3)	0.725
Overweight	1.8 (1.1, 2.7)	0.008	1.3 (0.8, 2.0)	0.353
Obese	4.6 (2.8, 7.5)	<0.001	3.0 (1.7, 5.2)	<0.001
**Parity**				
0	***Reference***		***Reference***	
1	1.2 (0.7, 1.9)	0.475	0.6 (0.4, 1.1)	0.142
2	1.1 (0.7, 1.9)	0.588	0.6 (0.3, 1.1)	0.096
3+	1.3 (0.8, 2.2)	0.225	0.4 (0.2, 0.9)	0.022
**Ethnic origin**				
White British	***Reference***		***Reference***	
Pakistani	0.7 (0.4, 1.0)	0.081	0.6 (0.3, 1.2)	0.135
Other	0.5 (0.3, 0.9)	0.020	0.4 (0.2, 0.8)	0.015
**Highest educational qualification**				
5 GCSE equivalent or less	***Reference***		***Reference***	
A-level equivalent	0.8 (0.5, 1.5)	0.554	0.7 (0.3, 1.3)	0.250
Higher than A-level	0.8 (0.5, 1.2)	0.219	0.7 (0.4, 1.2)	0.171
Other/Unknown	1.0 (0.5, 2.0)	0.996	0.7 (0.3, 1.6)	0.396
**Employment status**				
Currently employed	***Reference***		***Reference***	
Previously employed	1.4 (0.9, 2.2)	0.161	1.1 (0.6, 2.1)	0.649
Never employed	0.7 (0.5, 1.1)	0.139	0.7 (0.4, 1.3)	0.244
**Physical activity levels**				
Active	***Reference***		***Reference***	
Moderately active	0.7 (0.3, 1.9)	0.538	0.7 (0.2, 1.9)	0.467
Moderately inactive	1.1 (0.4, 2.8)	0.793	1.1 (0.4, 3.0)	0.905
Inactive	1.0 (0.4, 2.4)	0.919	1.1 (0.4, 2.9)	0.882
**Smoking during pregnancy**	2.6 (1.2, 5.5)	0.011	1.9 (0.8, 4.5)	0.140
**Family history of diabetes**	1.3 (0.9, 1.9)	0.156	1.2 (0.8, 1.9)	0.337
**Gestational age at OGTT (weeks)**	0.9 (0.8, 0.9)	0.004	0.9 (0.8, 1.0)	0.045
**Fasting glucose at OGTT (mmol/L)**	2.1 (1.6, 2.7)	<0.001	1.7 (1.3, 2.3)	<0.001
**2h post-load glucose at OGTT (mmol/L)**	1.5 (1.2, 1.7)	<0.001	1.4 (1.1, 1.7)	<0.001

A-level: UK highest qualification in high school; BMI: body mass index; GCSE: general certificate of secondary education (UK standard minimum level of education); OGTT: oral glucose tolerance test

For peer review only - http://bmjopen.bmj.com/site/about/guidelines.xhtml

Table 3 Maternal characteristics by GDM treatment type after metformin introduction (2009-2011)

	Lifestyle changes advice (n=123)	Insulin (n=200)	Metformin (n=73)	*p*	*p**	n (%) missing
**Age at childbirth† (years)**, median (IQR)	29.1 (7.8)	31.8 (8.2)	30.6 (9.0)	<0.001	0.004	0
**BMI at booking† (kg/m²)**, median (IQR)	24.7 (4.9)	28.1 (9.2)	29.3 (6.5)	<0.001	0.001	21 (2.8)
**BMI category at booking§** , n (%)				<0.001	<0.001	21 (2.8)
Underweight *(BMI<18.5 kg/m²)*	4 (3.4)	5 (2.7)	0			
Normal weight *(18.5≤BMI≤24.9 kg/m²)*	57 (48.3)	55 (29.3)	16 (23.2)			
Overweight *(25.0≤BMI≤29.9 kg/m²)*	40 (33.9)	51 (27.1)	22 (31.9)			
Obese *(BMI ≥30.0 kg/m²)*	17 (14.4)	77 (41.0)	31 (44.9)			
**Smoking during pregnancy‡**, n (%)				0.004	0.032	0
Yes	4 (3.3)	29 (14.5)	6 (8.2)			
No	119 (96.7)	171 (85.5)	67 (91.8)			
**Parity‡** n (%)				0.145	0.580	6 (0.8)
0	52 (43.0)	68 (34.5)	22 (30.6)			
1	26 (21.5)	46 (23.3)	21 (29.2)			
2	18 (14.9)	35 (17.8)	19 (26.4)			
3+	25 (20.7)	48 (24.4)	10 (13.9)			
**Physical activity levels‡** n (%)				0.424	0.630	1 (0.1)
Inactive	71 (57.7)	116 (58.3)	43 (58.9)			
Moderately inactive	23 (18.7)	43 (21.6)	9 (12.3)			
Moderately active	22 (17.9)	30 (15.1)	13 (17.8)			
Active	7 (5.7)	10 (5.0)	8 (11.0)			
**Ethnic group‡** n (%)				0.210	0.630	0
White British	24 (19.5)	54 (27.0)	15 (20.5)			
Pakistani	74 (60.2)	113 (56.5)	50 (68.5)			
Other	25 (20.3)	33 (16.5)	8 (11.0)			
**Highest educational qualification‡** n (%)				0.297	0.630	0
5 GCSE equivalent or less	53 (43.1)	104 (52.0)	32 (43.8)			
A-level equivalent	17 (13.8)	27 (13.5)	7 (9.6)			
Higher than A-level	44 (35.8)	55 (27.5)	31 (42.5)			
Other/Unknown	9 (7.3)	14 (7.0)	3 (4.1)			
**Family history of diabetes‡** n (%)				0.099	0.553	27 (3.5)
Yes	65 (57.0)	130 (69.1)	42 (62.7)			
No	49 (43.0)	58 (30.8)	25 (37.3)			
**Mother's employment status‡** n (%)				0.079	0.553	0
Currently employed	48 (39.0)	80 (40.0)	28 (38.4)			
Previously employed	19 (15.4)	54 (27.0)	20 (27.4)			
Never employed	56 (45.5)	66 (33.0)	25 (34.2)			
**Gestational age at OGTT† (weeks),** median (IQR)	26.3 (1.8)	26.1 (0.8)	26.3 (0.7)	0.087	0.553	8 (1.0)
**Fasting glucose concentrations at OGTT† (mmol/L),** median (IQR)	4.7 (0.8)	5.2 (1.2)	4.8 (0.7)	<0.001	0.001	8 (1.0)
**2h post-load glucose concentrations at OGTT† (mmol/L),** median (IQR)	8.2 (0.8)	8.6 (1.6)	8.4 (1.3)	<0.001	0.001	8 (1.0)

A-level: UK highest qualification in high school; BMI: body mass index; GCSE: general certificate of secondary education; OGTT: oral glucose tolerance test
Continuous data presented as median and interquartile range (IQR).
Categorical data presented as frequencies and percentages.
*Adjusted *P*-values after Holm-Bonferroni correction
†Kruskal-Wallis test
‡Chi-square test
§Fisher's exact test

For peer review only - http://bmjopen.bmj.com/site/about/guidelines.xhtml

Table 4 Associations between maternal characteristics and GDM pharmaceutical treatment after metformin introduction

	**Lifestyle changes advice**	**Insulin**		**Metformin**		**Insulin**	**Metformin**	
	Reference	*Adjusted RRR (95% CI)*	*p*	*Adjusted RRR (95% CI)*	*p*	***Reference***	*Adjusted RRR (95% CI)*	*p*
**Mother age at childbirth (years)**		1.1 (1.0, 1.2)	0.001	1.0 (1.0, 1.1)	0.160		0.9 (0.9. 1.0)	0.141
**BMI categories at booking (kg/m^2)**								
Normal weight		***Reference***		***Reference***			***Reference***	
Underweight		1.8 (0.4, 9.1)	0.444	-	-		-	-
Overweight		0.6 (0.3, 1.3)	0.221	1.6 (0.6, 4.0)	0.290		2.6 (1.0, 6.4)	0.044
Obese		2.3 (1.0, 5.2)	0.051	7.3 (2.7, 20.0)	<0.001		3.2 (1.3, 7.8)	0.010
**Parity**								
0		***Reference***		***Reference***			***Reference***	
1		0.6 (0.3, 1.4)	0.234	0.8 (0.3, 2.2)	0.704		1.3 (0.5, 3.3)	0.524
2		0.5 (0.2, 1.4)	0.204	1.1 (0.4, 3.2)	0.817		2.1 (0.8, 5.5)	0.143
3+		0.6 (0.2, 1.8)	0.398	0.4 (0.1, 1.6)	0.210		0.7 (0.2, 2.2)	0.521
**Ethnic origin**								
White British		***Reference***		***Reference***			***Reference***	
Pakistani		0.5 (0.2, 1.4)	0.197	1.7 (0.5, 5.5)	0.359		3.2 (1.1, 9.3)	0.031
Other		0.5 (0.2, 1.4)	0.187	0.7 (0.2, 2.6)	0.618		1.4 (0.4, 4.7)	0.605
**Highest educational qualification**								
5 GCSE equivalent or less		***Reference***		***Reference***			***Reference***	
A-level equivalent		0.6 (0.2, 1.6)	0.360	0.5 (0.1, 1.9)	0.325		0.8 (0.2, 2.7)	0.756
Higher than A-level		0.6 (0.3, 1.4)	0.271	1.0 (0.4, 2.6)	0.925		1.6 (0.7, 3.7)	0.281
Other/Unknown		0.8 (0.2, 2.4)	0.645	0.4 (0.08, 1.7)	0.208		0.5 (0.1, 2.1)	0.328
**Employment status**								
Currently employed		***Reference***		***Reference***			***Reference***	
Previously employed		1.6 (0.6, 3.9)	0.297	1.7 (0.6, 5.0)	0.306		1.1 (0.4, 2.8)	0.866
Never employed		0.8 (0.3, 1.9)	0.613	0.7 (0.2, 2.3)	0.617		0.9 (0.3, 2.7)	0.922
**Physical activity levels**								
Active		***Reference***		***Reference***			***Reference***	
Moderately active		0.5 (0.1, 2.0)	0.374	0.4 (0.09, 1.8)	0.231		0.7 (0.2, 2.9)	0.647
Moderately inactive		1.0 (0.3, 3.5)	0.957	0.2 (0.05, 1.1)	0.063		0.2 (0.05, 1.0)	0.051
Inactive		0.8 (0.2, 3.0)	0.809	0.5 (0.1, 2.1)	0.328		0.6 (0.1, 2.2)	0.405
**Smoking during pregnancy**		4.1 (1.0, 16.9)	0.048	1.9 (0.3, 11.7)	0.474		0.5 (0.1, 1.8)	0.270
**Family history of diabetes**		1.2 (0.6, 2.2)	0.554	1.0 (0.5, 2.1)	0.959		0.8 (0.4, 1.7)	0.652
**Gestational age at OGTT (weeks)**		0.9 (0.8, 1.1)	0.364	1.0 (0.8, 1.1)	0.747		1.0 (0.9, 1.2)	0.644
**Fasting glucose at OGTT (mmol/L)**		2.3 (1.5, 3.6)	<0.001	0.8 (0.4, 1.4)	0.456		0.3 (0.2, 0.6)	<0.001
**2h post-load glucose at OGTT (mmol/L)**		1.2 (0.9, 1.7)	0.127	1.2 (0.9, 1.8)	0.245		1.0 (0.7, 1.3)	0.968

BMI: body mass index; CI: confidence interval; OGTT: oral glucose tolerance test, RRR: relative risk ratio

For peer review only - http://bmjopen.bmj.com/site/about/guidelines.xhtml

Figure 1 legend: Flowchart of study participation

For peer review only

For peer review only - http://bmjopen.bmj.com/site/about/guidelines.xhtml

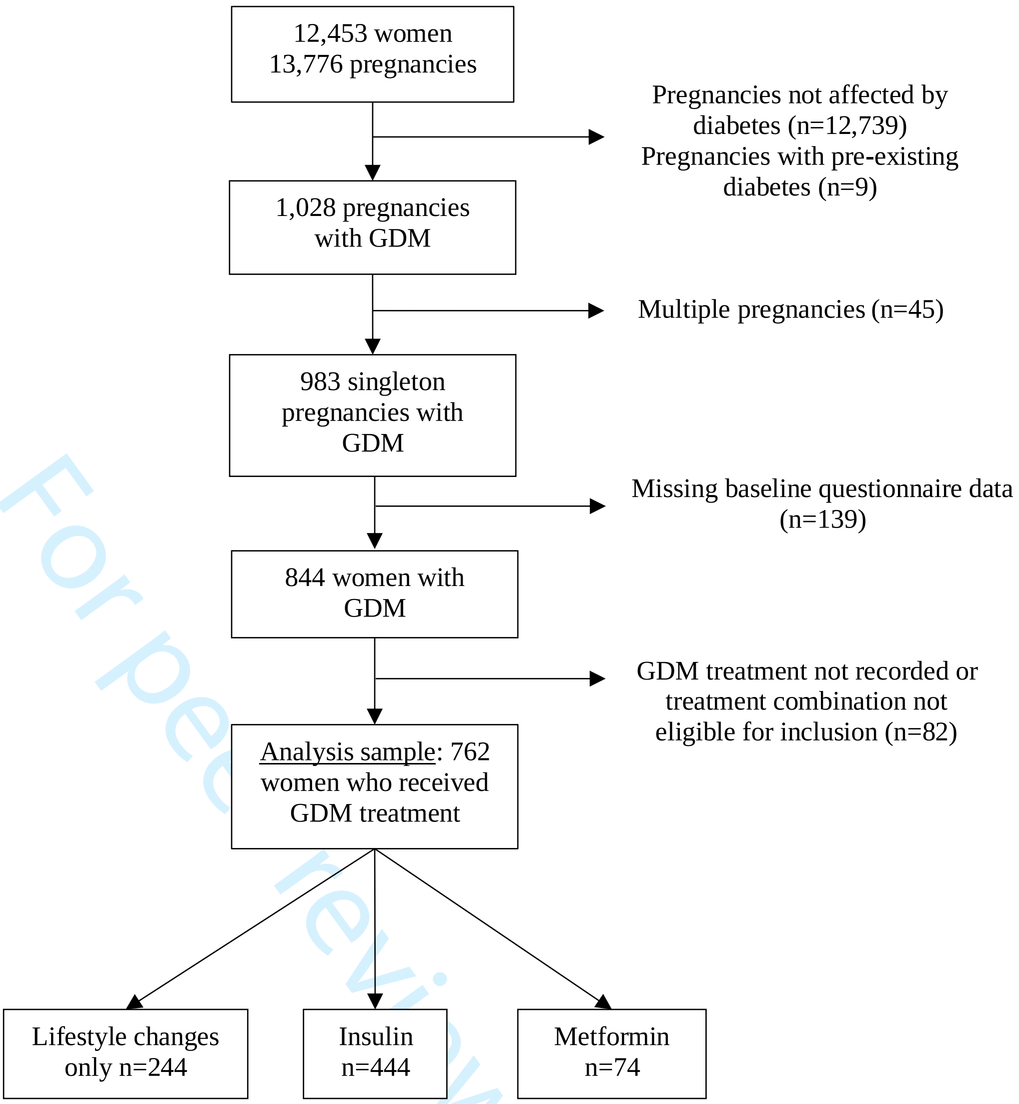

Figure 1 Flowchart of study participation

For peer review only

Supplementary Table 1 Maternal characteristics by maternal ethnicity

	**White British (n=142)**	**Pakistani (n=324)**
**GDM treatment**, n (%)		
Lifestyle changes advice alone	37 (26.1)	111 (34.3)
Pharmaceutical treatment	105 (73.9)	213 (65.7)
**Age at childbirth (years)**, median (IQR)	30.0 (9.4)	30.8 (7.4)
**BMI at booking (kg/m^2)**, median (IQR)	27.9 (10.5)	26.9 (7.1)
**BMI category at booking** , n (%)		
Underweight *(BMI<18.5 kg/m^2)*	2 (1.4)	7 (2.2)
Normal weight *(18.5≤BMI≤24.9 kg/m^2)*	53 (37.3)	99 (30.6)
Overweight *(25.0≤BMI≤29.9 kg/m^2)*	29 (20.4)	121 (37.3)
Obese *(BMI ≥30.0 kg/m^2)*	58 (40.8)	97 (29.9)
**Smoking during pregnancy**, n (%)		
Yes	39 (27.5)	10 (3.1)
No	103 (72.5)	314 (96.9)
**Parity,** n (%)		
0	68 (47.9)	94 (29.0)
1	43 (30.3)	63 (19.4)
2	20 (14.1)	62 (19.1)
3+	11 (7.7)	105 (32.4)
**Physical activity levels,** n (%)		
Inactive	53 (37.3)	254 (78.4)
Moderately inactive	37 (26.1)	41 (12.6)
Moderately active	37 (26.1)	21 (6.5)
Active	15 (10.6)	8 (2.5)
**Highest educational qualification,** n (%)		
5 GCSE equivalent or less	60 (42.2)	192 (59.3)
A-level equivalent	25 (17.6)	30 (9.3)
Higher than A-level	40 (28.2)	85 (26.2)
Other/Unknown	17 (12.0)	17 (5.2)
**Family history of diabetes,** n (%)		
Yes	75 (52.8)	221 (68.2)
No	67 (47.2)	103 (31.8)
**Mother's employment status,** n (%)		
Currently employed	105 (73.9)	65 (20.1)
Previously employed	31 (21.8)	90 (27.8)
Never employed	6 (4.2)	169 (52.2)
**Gestational age at OGTT (weeks),** median (IQR)	26.3 (1.1)	26.3 (1.1)
**Fasting glucose concentrations at OGTT (mmol/L),** median (IQR)	4.7 (0.8)	5.1 (0.9)
**2h post-load glucose concentrations at OGTT (mmol/L),** median (IQR)	8.3 (0.9)	8.6 (1.5)

A-level: UK highest qualification in high school; BMI: body mass index; GCSE: general certificate of secondary education; OGTT: oral glucose tolerance test
Continuous data presented as median and interquartile range (IQR).
Categorical data presented as frequencies and percentages.

For peer review only - http://bmjopen.bmj.com/site/about/guidelines.xhtml

Supplementary Table 2 Associations between maternal characteristics and GDM pharmaceutical treatment relative to lifestyle changes advice stratified by maternal ethnicity

	White British (N=142)		Pakistani (N=324)	
	Pharmaceutical treatment		Pharmaceutical treatment	
	Adjusted OR (95% CI)	*p*	*Adjusted OR (95% CI)*	*p*
**Mother age at childbirth (years)**	1.1 (1.0, 1.2)	0.145	1.1 (1.0, 1.2)	0.008
**BMI categories at booking (kg/m^2)**				
Normal weight	***Reference***		***Reference***	
Underweight	1.1 (0.03, 46.8)	0.943	1.2 (0.2, 7.0)	0.798
Overweight	1.0 (0.3, 3.4)	0.954	1.2 (0.6, 2.3)	0.546
Obese	2.1 (0.6, 7.0)	0.215	4.0 (1.8, 8.8)	0.001
**Parity**				
0	***Reference***		***Reference***	
1	0.7 (0.2, 2.3)	0.613	0.5 (0.2, 1.2)	0.157
2	0.6 (0.1, 3.5)	0.611	0.5 (0.2, 1.2)	0.152
3+	0.09 (0.01, 0.8)	0.027	0.6 (0.2, 1.4)	0.208
**Highest educational qualification**				
5 GCSE equivalent or less	***Reference***		***Reference***	
A-level equivalent	0.5 (0.1, 2.1)	0.361	0.8 (0.3, 2.2)	0.678
Higher than A-level	0.7 (0.2, 2.6)	0.577	0.6 (0.3, 1.1)	0.122
Other/Unknown	0.5 (0.1, 2.6)	0.417	1.1 (0.3, 3.8)	0.925
**Employment status**				
Currently employed	***Reference***		***Reference***	
Previously employed	0.5 (0.1, 2.0)	0.328	1.1 (0.4, 2.6)	0.862
Never employed	0.3 (0.02, 3.7)	0.343	0.6 (0.2, 1.3)	0.190
**Physical activity levels**				
Active	***Reference***		***Reference***	
Moderately active	1.3 (0.3, 5.7)	0.737	0.3 (0.05, 2.3)	0.281
Moderately inactive	1.7 (0.4, 7.5)	0.502	1.1 (0.2, 6.6)	0.912
Inactive	3.1 (0.7, 13.5)	0.134	0.9 (0.1, 4.9)	0.883
**Smoking during pregnancy**	2.1 (0.6, 7.4)	0.247	3.5 (0.4, 33.6)	0.269
**Family history of diabetes**	2.5 (1.0, 6.7)	0.058	1.0 (0.5, 1.8)	>0.999
**Gestational age at OGTT (weeks)**	0.9 (0.7, 1.2)	0.605	0.9 (0.8, 1.0)	0.070
**Fasting glucose at OGTT (mmol/L)**	2.5 (0.9, 6.5)	0.066	1.7 (1.1, 2.4)	0.008
**2h post-load glucose at OGTT (mmol/L)**	2.3 (1.0, 5.3)	0.039	1.2 (1.0, 1.5)	0.049

BMI: body mass index; CI: confidence interval; OGTT: oral glucose tolerance test

For peer review only - http://bmjopen.bmj.com/site/about/guidelines.xhtml

STROBE Statement—Checklist of items that should be included in reports of ***cohort studies***

	Item No	Recommendation	Reported on page #
**Title and abstract**	1	(*a*) Indicate the study's design with a commonly used term in the title or the abstract	1
		(*b*) Provide in the abstract an informative and balanced summary of what was done and what was found	2-3
**Introduction**			
Background/rationale	2	Explain the scientific background and rationale for the investigation being reported	5-6
Objectives	3	State specific objectives, including any prespecified hypotheses	6
**Methods**			
Study design	4	Present key elements of study design early in the paper	6
Setting	5	Describe the setting, locations, and relevant dates, including periods of recruitment, exposure, follow-up, and data collection	6-7
Participants	6	(*a*) Give the eligibility criteria, and the sources and methods of selection of participants. Describe methods of follow-up	7
		(*b*) For matched studies, give matching criteria and number of exposed and unexposed	N/A
Variables	7	Clearly define all outcomes, exposures, predictors, potential confounders, and effect modifiers. Give diagnostic criteria, if applicable	8-9
Data sources/ measurement	8*	For each variable of interest, give sources of data and details of methods of assessment (measurement). Describe comparability of assessment methods if there is more than one group	8-9
Bias	9	Describe any efforts to address potential sources of bias	10,11
Study size	10	Explain how the study size was arrived at	12
Quantitative variables	11	Explain how quantitative variables were handled in the analyses. If applicable, describe which groupings were chosen and why	10-11
Statistical methods	12	(*a*) Describe all statistical methods, including those used to control for confounding	10-11
		(*b*) Describe any methods used to examine subgroups and interactions	10-11
		(*c*) Explain how missing data were addressed	10-11
		(*d*) If applicable, explain how loss to follow-up was addressed	N/A
		(*e*) Describe any sensitivity analyses	11
**Results**			
Participants	13*	(a) Report numbers of individuals at each stage of study—eg numbers potentially eligible, examined for eligibility, confirmed eligible, included in the study, completing follow-up, and analysed	12
		(b) Give reasons for non-participation at each stage	12
		(c) Consider use of a flow diagram	Fig. 1
Descriptive data	14*	(a) Give characteristics of study participants (eg demographic, clinical, social) and information on exposures and potential confounders	12
		(b) Indicate number of participants with missing data for each variable of interest	Tables 1, 3
		(c) Summarise follow-up time (eg, average and total amount)	N/A
Outcome data	15*	Report numbers of outcome events or summary measures over time	12, 14
Main results	16	(*a*) Give unadjusted estimates and, if applicable, confounder-adjusted estimates and their precision (eg, 95% confidence interval). Make clear which	13-14

For peer review only - http://bmjopen.bmj.com/site/about/guidelines.xhtml

		confounders were adjusted for and why they were included	
		(*b*) Report category boundaries when continuous variables were categorized	13-14
		(*c*) If relevant, consider translating estimates of relative risk into absolute risk for a meaningful time period	N/A
Other analyses	17	Report other analyses done—eg analyses of subgroups and interactions, and sensitivity analyses	13
**Discussion**			
Key results	18	Summarise key results with reference to study objectives	14-15
Limitations	19	Discuss limitations of the study, taking into account sources of potential bias or imprecision. Discuss both direction and magnitude of any potential bias	19
Interpretation	20	Give a cautious overall interpretation of results considering objectives, limitations, multiplicity of analyses, results from similar studies, and other relevant evidence	15-18
Generalisability	21	Discuss the generalisability (external validity) of the study results	19
**Other information**			
Funding	22	Give the source of funding and the role of the funders for the present study and, if applicable, for the original study on which the present article is based	6, 21

*Give information separately for exposed and unexposed groups.

Note: An Explanation and Elaboration article discusses each checklist item and gives methodological background and published examples of transparent reporting. The STROBE checklist is best used in conjunction with this article (freely available on the Web sites of PLoS Medicine at http://www.plosmedicine.org/, Annals of Internal Medicine at http://www.annals.org/, and Epidemiology at http://www.epidem.com/). Information on the STROBE Initiative is available at http://www.strobe-statement.org.

For peer review only
